# Association of TIGIT and CD155 with KRAS, NRAS, BRAF, PIK3CA, and AKT Gene Mutations, MSI Status, and Cytokine Profiles in Colorectal Cancer

**DOI:** 10.3390/ijms27020937

**Published:** 2026-01-17

**Authors:** Błażej Ochman, Piotr Limanówka, Sylwia Mielcarska, Agnieszka Kula, Miriam Dawidowicz, Dorota Hudy, Monika Szrot, Jerzy Piecuch, Zenon Czuba, Dariusz Waniczek, Elżbieta Świętochowska

**Affiliations:** 1 Department of Medical and Molecular Biology, Faculty of Medical Sciences in Zabrze, Medical University of Silesia, 19 Jordana, 41-808 Zabrze, Poland; s82955@365.sum.edu.pl (P.L.); d201109@365.sum.edu.pl (S.M.); dorota.hudy@sum.edu.pl (D.H.); eswietochowska@sum.edu.pl (E.Ś.); 2 Department of Oncological Surgery, Faculty of Medical Sciences in Zabrze, Medical University of Silesia, 41-808 Katowice, Poland; d201070@365.sum.edu.pl (A.K.); d201069@365.sum.edu.pl (M.D.); dwaniczek@sum.edu.pl (D.W.); 3 Department of General and Bariatric Surgery and Emergency Medicine in Zabrze, Faculty of Medical Sciences in Zabrze, Medical University of Silesia, 10 Marii Curie-Skłodowskiej, 41-800 Zabrze, Poland; mszrot@sum.edu.pl (M.S.); jpiecuch@sum.edu.pl (J.P.); 4 Department of Microbiology and Immunology, Faculty of Medical Sciences in Zabrze, Medical University of Silesia, 19 Jordana, 41-808 Zabrze, Poland; zczuba@sum.edu.pl

**Keywords:** colorectal neoplasms, TIGIT, CD155, PVR, microsatellite instability (MSI), tumor immune microenvironment, KRAS, NRAS, PIK3CA, BRAF

## Abstract

TIGIT and its ligand CD155 (PVR) are emerging immune checkpoints in colorectal cancer (CRC), but their associations with mutational subtypes and the tumor immune milieu remain unclear. We quantified TIGIT and CD155 proteins by ELISA in paired CRC tumors and matched surgical margins (*n* = 131) and evaluated associations with clinicopathological features, MSI status, and *KRAS/NRAS/BRAF/PIK3CA/AKT1* mutations (*n* = 104). Both TIGIT and CD155 were significantly elevated in tumor tissue versus margins (*p* < 0.0001) and showed no association with TNM stage, clinical stage, grade, or tumor location. TIGIT levels were higher in MSI than MSS tumors and in *BRAF*-mutant compared to *BRAF* wild-type tumors, while CD155 expression showed no consistent MSI- or mutation-dependent differences. Cytokine profiling identified IFN-g as the only shared positive associate of TIGIT and CD155; CD155 additionally associated with TRAIL, IL-1Ra, M-CSF, and PDGF-bb. In external transcriptomic validation (TCGA-CRC), GSEA indicated enrichment of interferon/inflammatory programs in TIGIT-high tumors, while CD155-high tumors preferentially showed proliferation-related MYC/E2F/G2M signatures. Together, these findings support tumor-wide upregulation of the TIGIT/CD155 axis in CRC and suggest that TIGIT, more than CD155, tracks with MSI/*BRAF*-associated immune activation, providing a rationale for patient stratification in checkpoint-directed immunotherapy.

## 1. Introduction

TIGIT (T cell immunoreceptor with Ig and ITIM domains) and its ligand CD155 (PVR) function as immune checkpoint molecules [1]. TIGIT is an inhibitory receptor expressed on the surface of distinct immune cell subsets, mainly T cells, such as CD8^+^, CD4^+^, and regulatory T cells (Tregs), and also Natural Killer (NK) cells [1,2,3]. TIGIT primarily interacts with four ligands, among which CD155 is considered the dominant binding partner [4,5]. CD155 is a nectin-like adhesion molecule, lowly expressed at endothelial, epithelial, and immune cells [6,7]. CD155 expression can be markedly increased in specific settings, including stimulation by inflammatory cytokines, cellular stress, heightened proliferative activity, and conditions present within the tumor microenvironment (TME) [8,9,10]. Through multiple downstream mechanisms, the TIGIT/CD155 interaction suppresses the function of T cells and NK cells, thereby attenuating antitumor immunity and promoting tumor development and progression [11,12]. Accordingly, therapeutic blockade of the TIGIT/CD155 axis is being actively investigated to restore effective antitumor immunity, improve responses to existing immune checkpoint inhibitors, and increase the number of patients who benefit from immunotherapy [13]. However, CD155 is not exclusively linked to the inhibitory TIGIT pathway. It also binds DNAM-1 (CD226), an activating receptor expressed on NK and T cells that promotes antitumor effector functions, as well as CD96 (TACTILE), which is commonly described as inhibitory but may exert activating or context-dependent effects in selected settings [14,15]. Collectively, signaling through the CD96/TIGIT/CD226 receptor axis reflects a dynamic balance between inhibitory and activating cues in NK and T cells, shaped by competing receptor–ligand interactions and reciprocal receptor cross-regulation within the TME [16].

Colorectal cancer (CRC) is characterized by a complex pathogenic landscape, with diverse genetic alterations that differentially shape the TME, including the density and composition of tumor-infiltrating immune cell subsets, local cytokine and chemokine profiles, and the expression of immunoregulatory molecules [17]. These molecular features, including mismatch repair (MMR) status, *RAS/BRAF* mutations, and other defining alterations, strongly influence sensitivity to systemic therapies, particularly immunotherapy [18,19,20]. In CRC, immune checkpoint blockade targeting PD-1/PD-L1 and CTLA-4 has demonstrated clinical efficacy mainly in tumors with high microsatellite instability and deficient mismatch repair (MSI-H/dMMR) [21,22]. However, MSI-H/dMMR tumors account for only approximately 10–15% of all CRC cases, and both primary and acquired resistance to checkpoint inhibition are common [23,24]. As a result, only a minority of patients currently gain durable benefit from approved immunotherapies. This gap underscores the need to broaden immunotherapeutic strategies beyond established checkpoints and to identify novel targets and combinations that can overcome therapeutic resistance [24]. In this context, inhibitory pathways such as TIGIT/CD155 are of particular interest, as they may modulate responses to PD-1/PD-L1 blockade and provide additional opportunities to reinvigorate antitumor immunity. As clinical development of TIGIT/CD155-directed therapies progresses, a more precise understanding of how TIGIT and CD155 expression relates to clinically relevant mutations in CRC, including *KRAS*, *NRAS*, *BRAF*, and *PIK3CA* mutations, and to key features of the TME will be crucial for the implementation of personalized treatment strategies.

*KRAS*, *NRAS*, *BRAF*, and *PIK3CA* mutations occur at different frequencies in CRC and exert distinct effects on tumor biology, including the composition and functional properties of the TME, particularly its immune component. Oncogenic mutations in *KRAS* occur in ~35–45% of CRC cases, whereas *NRAS* mutations are found in ~5–10% [25,26]. From a clinical perspective, *KRAS* and *NRAS* represent the most relevant members of the RAS family, which regulate key signaling cascades controlling cell proliferation, differentiation, adhesion, programmed cell death, and migration. Beyond their well-established oncogenic functions, *KRAS* mutations can also reshape the immune TME, promoting an immunosuppressive TME through multiple mechanisms [27,28,29]. *BRAF* mutations are present in about 10% of CRCs, more than 90% of which are accounted for by the *BRAF* V600E variant. *BRAF*-mutant CRCs frequently co-occur with MSI-H/dMMR status, increased expression of immune checkpoint-related genes, and an altered immune infiltrate characterized by higher densities of certain immune cell populations [30,31,32,33]. *PIK3CA* mutations occur in approximately 15–20% of CRC cases. *PIK3CA* mutation or overexpression has been associated with resistance to immunotherapy [34,35]. Collectively, *KRAS*, *NRAS*, *BRAF*, and *PIK3CA* mutations, together with MSI status, differentially modulate intracellular signaling networks and the immunological and stromal composition of the TME, thereby influencing prognosis and shaping therapeutic opportunities. In line with this, the NCCN Clinical Practice Guidelines in Oncology advise evaluating mutations in *KRAS*, *NRAS*, and *BRAF*, as well as MSI status, to enable personalized therapy selection and improve patient stratification in CRC [36].

Given the pivotal impact of these molecular alterations in defining the CRC immune landscape, it is likely that they also shape the activity of additional checkpoint axes beyond the canonical PD-1/PD-L1 and CTLA-4 pathways. In particular, the TIGIT/CD155 axis has gained attention as a critical regulator of T cell and NK cell function within the TME and as a promising target for next-generation immunotherapeutic approaches. The genomic background of CRC, including alterations in *KRAS*, *NRAS*, *PIK3CA*, *AKT1*, *BRAF*, and the tumor’s MSI status, may modulate both the abundance and biological relevance of the immune regulators TIGIT and CD155, thereby affecting immune evasion and clinical outcome. Accordingly, this study quantified TIGIT and CD155 expression in CRC specimens and paired adjacent non-malignant mucosa, and examined how their expression relates to the examined major oncogenic mutations and MSI status. In addition, we assessed the associations between TIGIT/CD155 levels and a set of chosen 48 cytokines, chemokines, and growth factors measured in tumor tissue to clarify how this checkpoint axis is integrated into the CRC TME and to identify potential biomarkers to support personalized immunotherapeutic strategies.

## 2. Results

### 2.1. TIGIT and CD155 Protein Concentrations Are Significantly Elevated in CRC Tissues Compared to Surgical Margins

**Figure 1:**
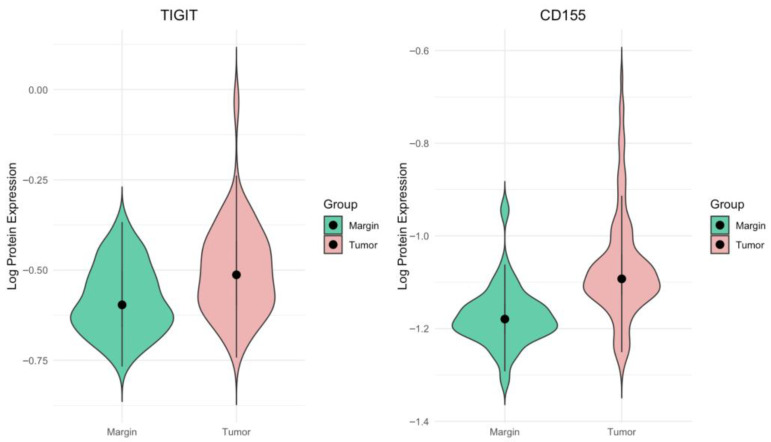
TIGIT and CD155 protein expression in CRC tissues and adjacent margin tissues. Violin plots. Wilcoxon signed-rank test *p*-value < 0.0001. All data were subjected to log10 transformation prior to analysis.

A total of 131 paired samples of CRC tissue specimens and surgical margin tissues were homogenized, and the concentrations of TIGIT and CD155 proteins were subsequently measured using ELISA. We observed significantly higher TIGIT and CD155 (PVR) protein concentration in CRC tumors compared with matched surgical margins (*p* < 0.0001) (Figure 1). To confirm these findings, we have conducted linear mixed-effects models with tissue type as a fixed effect and patient ID as a random intercept to account for within-patient dependence (Supplementary Table S1A,B). The models confirmed a significant increase in tumor samples for both TIGIT (tumor vs. margin: β = 0.0803; SE = 0.0097; df = 130.00; *p* < 0.0001) and CD155 (tumor vs. margin: β = 0.1052; SE = 0.0101; df = 128.94; *p* < 0.0001). These results were consistent with the paired Wilcoxon signed-rank tests, which also showed significant tumor-margin differences for TIGIT (*p* < 0.0001 and CD155 (*p* < 0.0001).

### 2.2. Associations of TIGIT and CD155 Protein Levels with Clinicopathological Features and MSI Status

**Figure 2:**
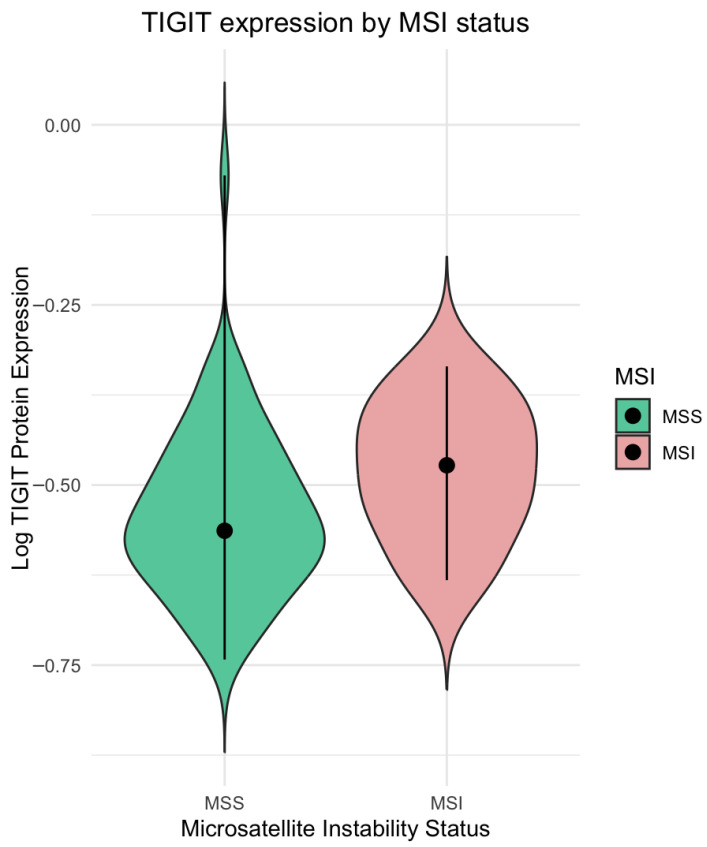
TIGIT protein concentration in CRC in groups with microsatellite instability (MSI) status compared to the microsatellite stable (MSS) tumor group. Violin plot. Mann–Whitney U test indicated a highly significant difference, with a *p*-value < 0.05.

We examined the associations between clinicopathological characteristics and the intratumoral levels of TIGIT and CD155 proteins in our study cohort. A comprehensive clinical profile of the patient group is presented in Table 1 (Methods). The analyzed variables included TNM classification, overall clinical stage, tumor grade, primary tumor localization (right- vs. left-sided), tumor-infiltrating lymphocytes (TILs), and microsatellite instability (MSI) status. No statistically significant associations were identified between TIGIT or CD155 expression and the parameters T, N, M, overall tumor stage, histological grade, or tumor localization. This lack of associations may be due to ELISA measurements in whole-tissue homogenates, which capture aggregate protein abundance across tumor, stromal, and immune compartments and may, therefore, mask compartment-specific, stage-related effects within a heterogeneous tumor microenvironment. TIGIT expression was significantly elevated in MSI tumors compared to MSS cases (*p* < 0.05) (Figure 2). In contrast, the expression levels of CD155 did not differ significantly between the MSI (*n* = 14) and MSS (*n* = 65) subgroups (*p* > 0.05). Full statistical output is available in Supplementary Tables S2 and S3. Based on these findings, neither TIGIT nor CD155 protein levels exhibited prognostic significance in the studied cohort.

### 2.3. Associations Between TIGIT and CD155 Expression and KRAS, NRAS, BRAF, PIK3CA, and AKT1 Mutations

Somatic mutations in the *KRAS*, *NRAS*, *BRAF*, *PIK3CA*, and *AKT1* genes were analyzed by real-time PCR. Mutation analysis was performed in a randomly selected subset of 104 CRC tissue samples drawn from a larger cohort of 131 CRC patients. *KRAS* was the most frequently mutated gene in the panel, with variants identified in 36.53% of the analyzed cases (*n* = 38). The predominant mutation hotspot was located at codons 12 and 13, accounting for 76.31% (*n* = 29) of all *KRAS* mutations and 27.88% of the entire patient cohort. *NRAS* mutations were present in 12.50% (*n* = 13) of samples, while *PIK3CA* and *BRAF* alterations were each detected in 6.73% (*n* = 7) of cases. Among the *PIK3CA*-mutated tumors, the 542/545 hotspot was identified in 85.71% (*n* = 6) of cases. The *BRAF* mutations detected in this study were located in exon 15 and included the following variants: V600E, V600E2, V600D, and V600K. A single *AKT1* mutation was found, representing 0.943% of the cohort. Notably, 9.62% of tumors (*n* = 10) harbored mutations in more than one of the examined genes. The most frequent co-mutation involved *KRAS* and *PIK3CA*, observed in 3.85% (*n* = 4) of tumors, followed by concurrent *KRAS* and *NRAS* mutations in 2.88% (*n* = 3) of cases. Rare combinations included *KRAS* with *BRAF*, *KRAS* with *AKT1*, and a triple mutation involving *KRAS*, *NRAS*, and *BRAF*, each detected in 0.96% (*n* = 1) of the study group. A detailed summary of mutation frequencies and case numbers is provided in Table 2.

**Figure 3:**
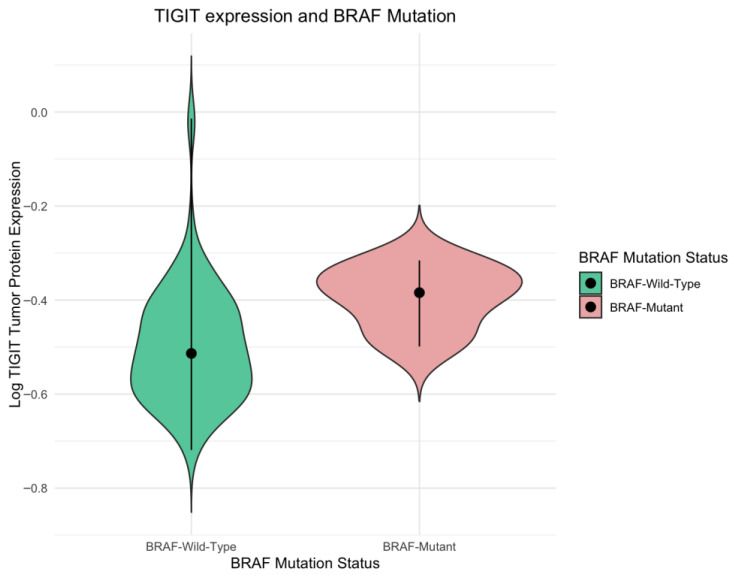
TIGIT expression in *BRAF*-mutated tumor group compared to BRAF wild-type CRC tumors. Violin plot. Mann–Whitney U test *p*-value < 0.05.

**Figure 4:**
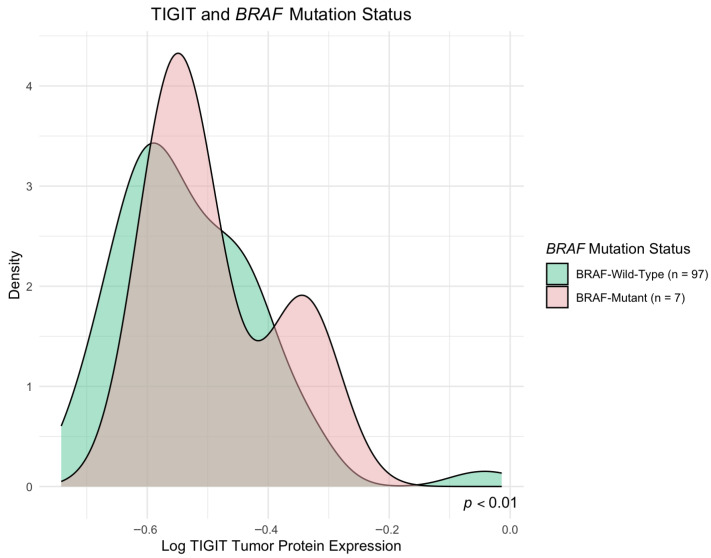
Density plot of TIGIT tumor protein expression according to *BRAF* mutation status. TIGIT expression differed significantly between groups (*p* < 0.05).

We found a significant increase in expression of TIGIT protein in *BRAF*-mutated CRC tumors compared to *BRAF* wild-type tumors (*p* < 0.01). TIGIT protein concentration, depending on *BRAF* mutation status, is presented in Figure 3. Figure 4 presents a density plot illustrating the distribution of TIGIT protein expression levels according to *BRAF* mutation status. In contrast, TIGIT protein levels did not differ significantly according to the mutation status of *KRAS*, *NRAS*, *PIK3CA*, or *AKT1* genes (*p* > 0.05). Similarly, CD155 concentrations were not significantly associated with the mutations of examined genes, with the exception of elevated CD155 expression in a small subset of tumors carrying the *KRAS* codon 146 mutation (*n* = 2) compared to *KRAS*-146 wild-type tumors (*p* < 0.05). Given the limited number of tumors with concurrent mutations in multiple genes, we also compared TIGIT and CD155 expression levels in this group with tumors lacking any detectable mutations in *KRAS*, *NRAS*, *BRAF*, *PIK3CA*, and *AKT1*. TIGIT or CD155 levels did not differ significantly between these groups (*p* > 0.05). A complete summary of the statistical analyses evaluating the relationship between gene mutation status and TIGIT/CD155 expression is provided in Supplementary Table S4.

### 2.4. TIGIT and CD155 Associations with Cytokines, Chemokines, and Growth Factor Expression in CRC TME

**Figure 5:**
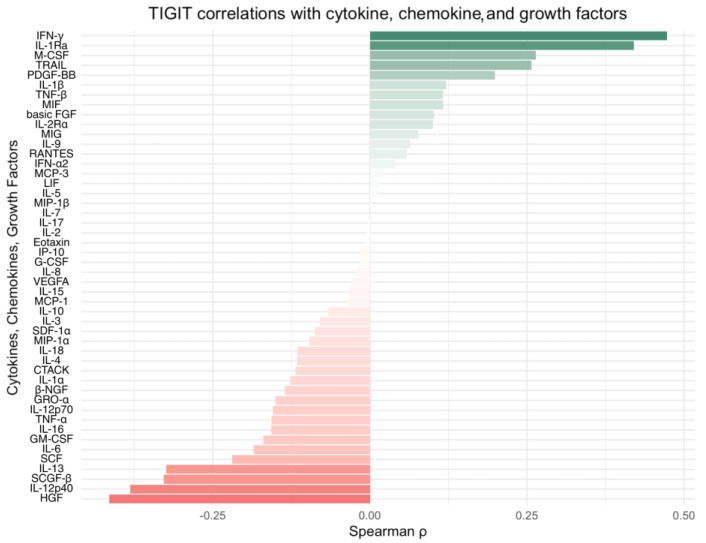
Spearman’s rank correlations between TIGIT tumor protein expression and examined cytokines, chemokines, and growth factors. Horizontal bar plots Spearman’s correlation coefficient (ρ), ordered by effect size; positive and negative associations are shown in green and red, respectively. Abbreviations: IFN-g, interferon gamma; IL-1Ra, interleukin-1 receptor antagonist; M-CSF, macrophage colony-stimulating factor; TRAIL, TNF-related apoptosis-inducing ligand; PDGF-BB, platelet-derived growth factor BB; IL-1β, interleukin 1 beta; TNF-β, tumor necrosis factor beta (lymphotoxin-a); MIF, macrophage migration inhibitory factor; basic FGF (bFGF/FGF2), basic fibroblast growth factor; IL-2Ra, interleukin-2 receptor alpha (CD25); MIG (CXCL9), monokine induced by gamma interferon; IL-9, interleukin 9; RANTES (CCL5), regulated upon activation, normal T cell expressed and secreted; IFN-a2, interferon alpha 2; MCP-3 (CCL7), monocyte chemoattractant protein 3; LIF, leukemia inhibitory factor; IL-5, interleukin 5; MIP-1β (CCL4), macrophage inflammatory protein 1 beta; IL-7, interleukin 7; IL-17, interleukin 17; IL-2, interleukin 2; Eotaxin (CCL11), eotaxin; IP-10 (CXCL10), interferon gamma-induced protein 10; G-CSF, granulocyte colony-stimulating factor; IL-8 (CXCL8), interleukin 8; VEGF-A, vascular endothelial growth factor A; IL-15, interleukin 15; MCP-1 (CCL2), monocyte chemoattractant protein 1; IL-10, interleukin 10; IL-3, interleukin 3; SDF-1a (CXCL12), stromal cell-derived factor 1 alpha; MIP-1a (CCL3), macrophage inflammatory protein 1 alpha; IL-18, interleukin 18; IL-4, interleukin 4; CTACK (CCL27), cutaneous T cell-attracting chemokine; IL-1a, interleukin 1 alpha; β-NGF, beta nerve growth factor; GRO-a (CXCL1), growth-related oncogene alpha; IL-12p70, interleukin 12 p70; TNF-a, tumor necrosis factor alpha; IL-16, interleukin 16; GM-CSF, granulocyte-macrophage colony-stimulating factor; IL-6, interleukin 6; SCF, stem cell factor (KIT ligand); IL-13, interleukin 13; SCGF-β (CLEC11A), stem cell growth factor beta; IL-12p40, interleukin 12 p40 subunit; HGF, hepatocyte growth factor.

**Figure 6:**
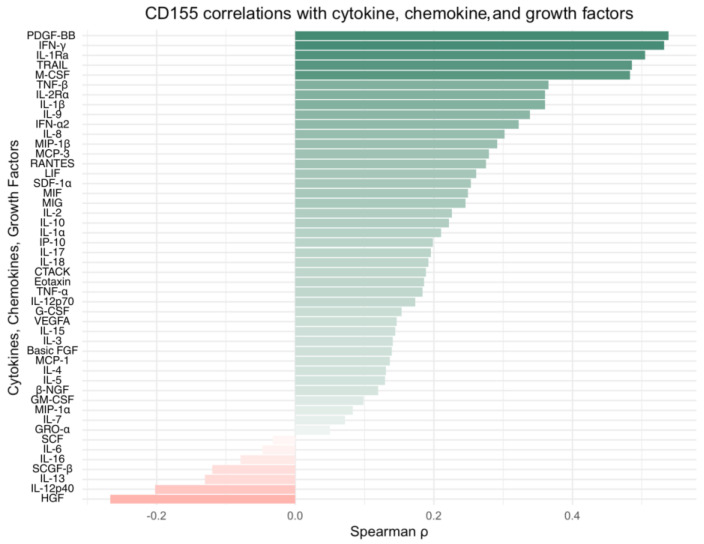
Spearman’s rank correlations between CD155 tumor protein expression and selected cytokines, chemokines, and growth factors. Horizontal bar plots indicate Spearman’s correlation coefficient (ρ), ranked by effect size; positive and negative associations are shown in green and red, respectively. Abbreviations: PDGF-BB, platelet-derived growth factor BB; IFN-g, interferon gamma; IL-1Ra, interleukin-1 receptor antagonist; TRAIL, TNF-related apoptosis-inducing ligand; M-CSF, macrophage colony-stimulating factor; TNF-β, tumor necrosis factor beta (lymphotoxin-a); IL-2Ra, interleukin-2 receptor alpha (CD25); IL-1β, interleukin 1 beta; IL-9, interleukin 9; IFN-a2, interferon alpha 2; IL-8 (CXCL8), interleukin 8; MIP-1β (CCL4), macrophage inflammatory protein 1 beta; MCP-3 (CCL7), monocyte chemoattractant protein 3; RANTES (CCL5), regulated upon activation, normal T cell expressed and secreted; LIF, leukemia inhibitory factor; SDF-1a (CXCL12), stromal cell-derived factor 1 alpha; MIF, macrophage migration inhibitory factor; MIG (CXCL9), monokine induced by gamma interferon; IL-2, interleukin 2; IL-10, interleukin 10; IL-1a, interleukin 1 alpha; IP-10 (CXCL10), interferon gamma-induced protein 10; IL-17, interleukin 17; IL-18, interleukin 18; CTACK (CCL27), cutaneous T cell-attracting chemokine; Eotaxin (CCL11), eotaxin; TNF-a, tumor necrosis factor alpha; IL-12p70, interleukin 12 p70; G-CSF, granulocyte colony-stimulating factor; VEGF-A, vascular endothelial growth factor A; IL-15, interleukin 15; IL-3, interleukin 3; basic FGF (bFGF/FGF2), basic fibroblast growth factor; MCP-1 (CCL2), monocyte chemoattractant protein 1; IL-4, interleukin 4; IL-5, interleukin 5; β-NGF, beta nerve growth factor; GM-CSF, granulocyte-macrophage colony-stimulating factor; MIP-1a (CCL3), macrophage inflammatory protein 1 alpha; IL-7, interleukin 7; GRO-a (CXCL1), growth-related oncogene alpha; SCF, stem cell factor (KIT ligand); IL-6, interleukin 6; IL-16, interleukin 16; SCGF-β (CLEC11A), stem cell growth factor beta; IL-13, interleukin 13; IL-12p40, interleukin 12 p40 subunit; HGF, hepatocyte growth factor.

To investigate the associations between TIGIT and CD155 protein levels and the cytokine profile within the CRC TME, we quantified protein concentration of 48 cytokines, chemokines, and growth factors and performed correlation analyses. Among the analyzed mediators, only a limited number of statistically significant associations were identified between TIGIT/CD155 and the measured cytokines, chemokines, and growth factors. CD155 levels showed positive correlations with TRAIL (*p* < 0.05, R = 0.486), IFN-g (*p* < 0.05, R = 0.532), IL-1Ra (*p* < 0.05, R = 0.505), M-CSF (*p* < 0.05, R = 0.483), and PDGF-bb (*p* < 0.05, R = 0.539). For TIGIT, a significant correlation was observed exclusively with IFN-g expression (*p* < 0.05, R = 0.473). Other correlations, including that with HGF expression, approached but did not reach statistical significance (*p* > 0.05). The ranked correlation bar plots showing Spearman’s rank correlations between TIGIT and CD155 protein expression and the examined cytokines, chemokines, and growth factors are presented in Figure 5 and Figure 6, respectively. All the results of the correlation analysis are demonstrated in Supplementary Material Table S5.

**Figure 7:**
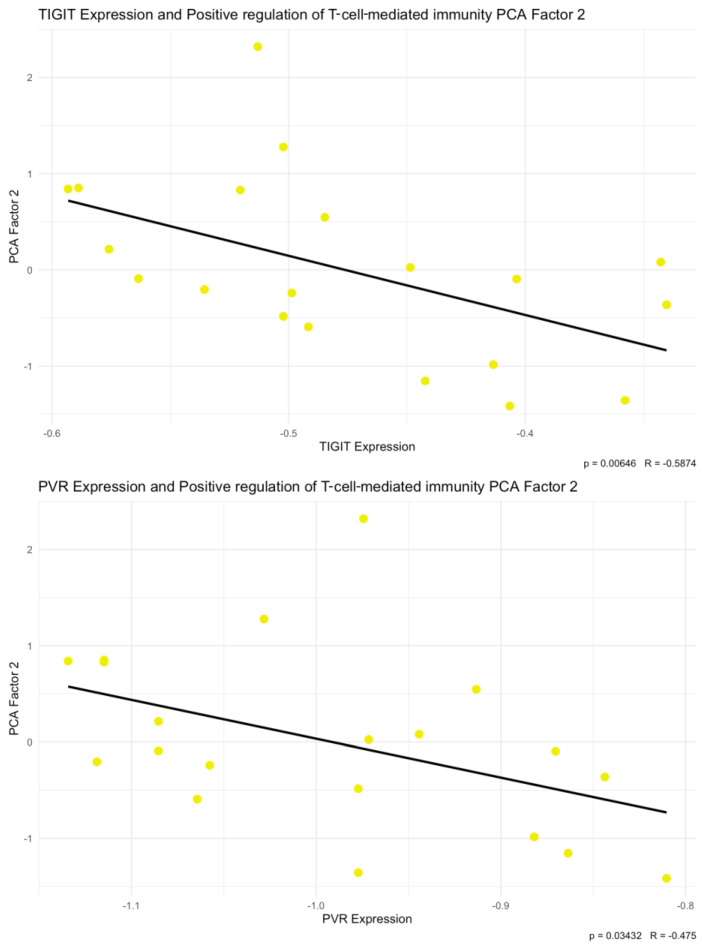
Correlations between TIGIT and CD155 (PVR) with positive regulation of T cell-mediated immunity set of cytokines PCA Factor 2 (*p* < 0.01, R = −0.5874, and *p* < 0.05, R = −0.475, respectively). The yellow dots indicate the PCA-derived data points used to assess the correlation between PCA Factor 2 and TIGIT/CD155 protein expression levels.

**Figure 8:**
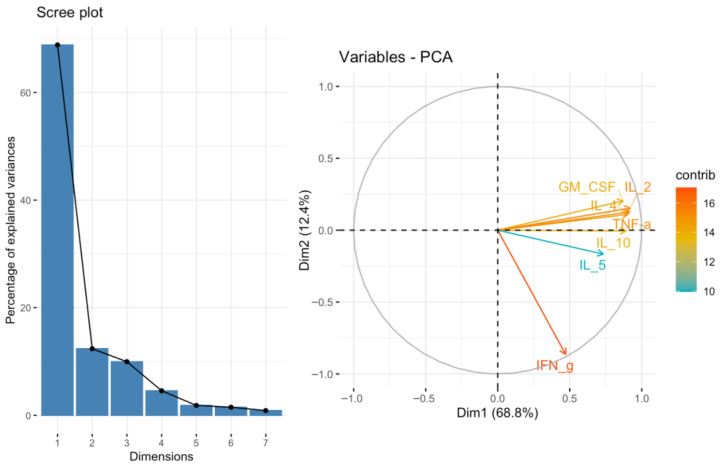
Scree plot and biplot from principal component analysis (PCA) for positive regulation of T cell-mediated immunity set of cytokines.

**Figure 9:**
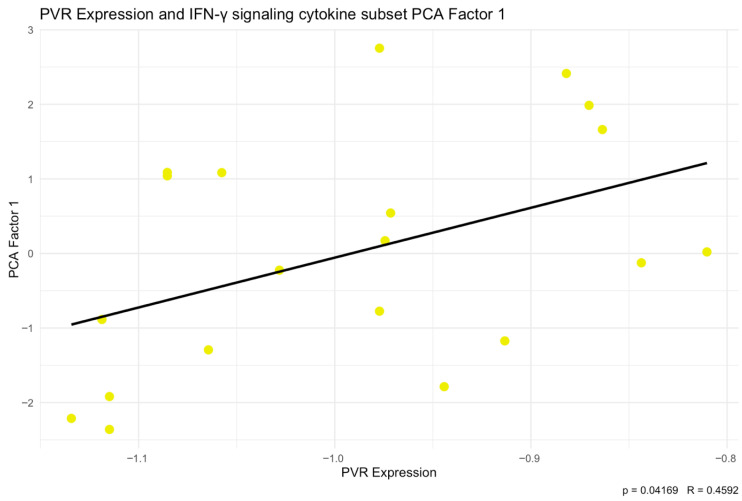
Correlations between TIGIT with Type II interferon signaling set of cytokines PCA Factor 1 (*p* < 0.05, R = 0.4592). The yellow dots indicate the PCA-derived data points used to assess the correlation between PCA Factor 1 and TIGIT protein expression levels.

**Figure 10:**
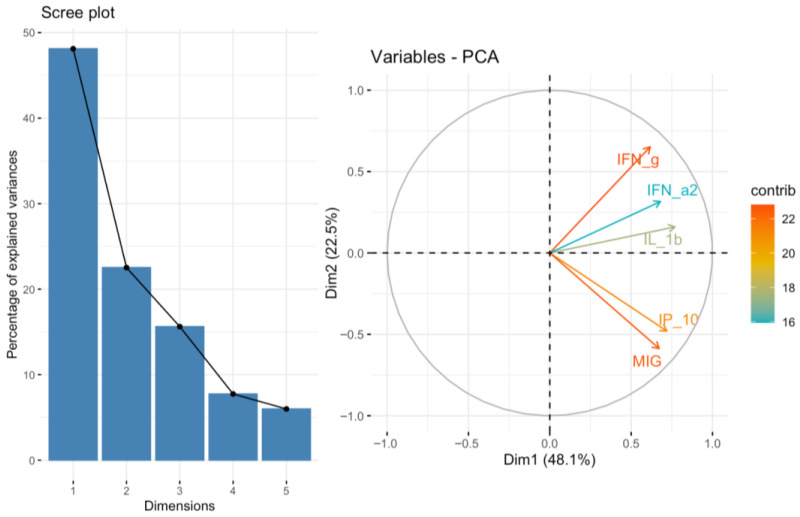
Scree plot and biplot from principal component analysis (PCA) for Type II interferon signaling set of cytokines.

To further characterize the correlations between TIGIT/CD155 axis protein expression and the selected cytokines, chemokines, and growth factors, we additionally performed principal component analysis (PCA). PCA was conducted for 14 predefined subsets of cytokines, chemokines, and growth factors. TIGIT and CD155 levels were subsequently correlated with the corresponding PCA factors. Among the analyzed subsets, we identified a significant negative correlation between both TIGIT (*p* < 0.01, R = −0.5874) and CD155 (*p* < 0.05, R = −0.475) expression and PCA Factor 2 for the cytokine subset associated with the GO term positive regulation of T cell-mediated immunity (Figure 7). The scree plot and biplot for this PCA are shown in Figure 8. Importantly, this factor was strongly driven by IFN-g (loading −0.864), indicating that the observed factor association is directionally consistent with the positive TIGIT/CD155–IFN-g correlations seen in the single-analyte analysis. In addition, TIGIT expression correlated positively with PCA Factor 1 from the Type II interferon signaling cytokine subset (*p* < 0.05, R = 0.4592) (Figure 9). The corresponding scree plot and biplot are shown in Figure 10. Eigenvalues and variable coordinates for examined subsets are provided in Table 3, Table 4, Table 5 and Table 6.

Collectively, these results support that higher TIGIT (and partly CD155) levels occur in an IFN-g-high, immune-inflamed TME, where inflammatory activation can coexist with compensatory upregulation of inhibitory checkpoint pathways, which may be linked to T cell dysfunction/exhaustion within the TME. Moreover, CD155 additionally correlated with mediators related to myeloid/stromal regulation (M-CSF, PDGF-bb, TRAIL) and counter-regulatory inflammation (IL-1Ra), suggesting that CD155 levels may capture broader tumor–stroma–myeloid remodeling rather than a purely T cell-restricted program. PCA recapitulated the single-correlate results by grouping IFN-g–linked mediators into a shared interferon-related component, with TIGIT showing the strongest alignment with this component, whereas CD155 displayed a broader association pattern spanning interferon-related and growth-factor/myeloid-related mediators.

### 2.5. GSEA of Hallmark Pathways in CRC According to TIGIT and CD155 Expression

**Figure 11:**
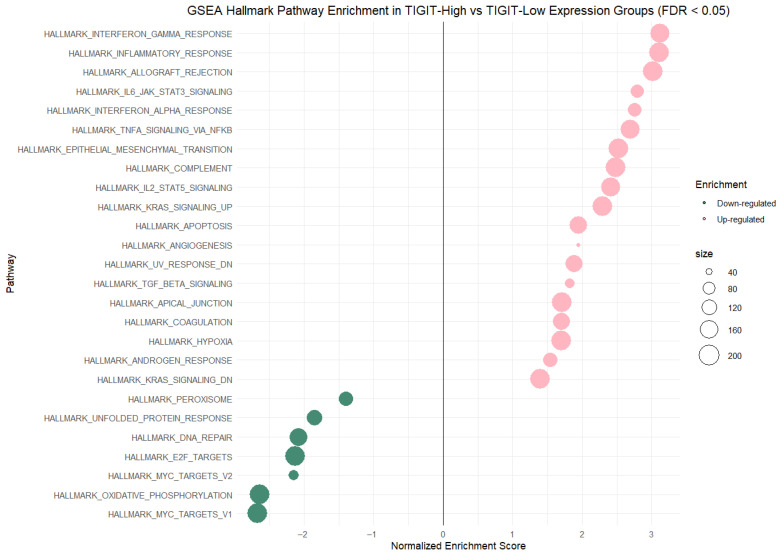
Gene set enrichment analysis (GSEA) of Hallmark pathways comparing TIGIT-high versus TIGIT-low expression groups (FDR < 0.05). Dots represent normalized enrichment scores (NESs) for each pathway, with point size proportional to gene set size.

To better characterize the biological and molecular programs, including signaling pathways, associated with TIGIT and CD155 expression, we conducted gene set enrichment analysis (GSEA). GSEA was also conducted to extend our findings on the associations between TIGIT/CD155 expression and the measured cytokines, chemokines, and growth factors. For the analysis, available samples were stratified into high- and low-expression groups for TIGIT or CD155, and enrichment scores were calculated. GSEA of Hallmark gene sets comparing TIGIT-high vs. TIGIT-low expression groups (FDR < 0.05) demonstrated a pronounced, bidirectional enrichment pattern, where positive NES values indicate enrichment in the TIGIT-high group and negative NES values indicate enrichment in the TIGIT-low group. The TIGIT-high group was strongly enriched for immune and inflammatory programs, led by Hallmark Interferon Gamma Response (NES = 3.12), and Hallmark Inflammatory Response (NES = 3.11), with additional enrichment of cytokine-associated signaling including Hallmark IL6-JAK-STAT3 Signaling (NES = 2.80), Hallmark Interferon Alpha Response (NES = 2.76), and Hallmark TNF-a Signaling via NF-KB (NES = 2.69). TIGIT-high tumors also showed enrichment of broader microenvironmental/remodeling signatures such as Hallmark Epithelial–Mesenchymal Transition (NES = 2.52), Hallmark Complement (NES = 2.49), with more moderate positive enrichment for hypoxia (NES = 1.70), and apical junction (NES = 1.72). In contrast, the TIGIT-low group was enriched for proliferation and metabolic pathways, including Hallmark MYC Targets V1 (NES = −2.67), Hallmark Oxidative Phosphorylation (NES = −2.63), Hallmark MYC Targets V2 (NES = −2.15), Hallmark E2F Targets (NES = −2.12), Hallmark DNA Repair (NES = −2.08), and Hallmark Unfolded Protein Response (NES = −1.85), as well as Hallmark Peroxisome (NES = −1.40). Overall, 26 Hallmark pathways met the significance threshold (FDR < 0.05), of which 19 were enriched in TIGIT-high (NES > 0) and 7 were enriched in TIGIT-low (NES < 0). The GSEA result plot for TIGIT gene expression is presented in Figure 11, and a table with detailed statistical outcomes is presented in Supplementary Material (Table S6).

**Figure 12:**
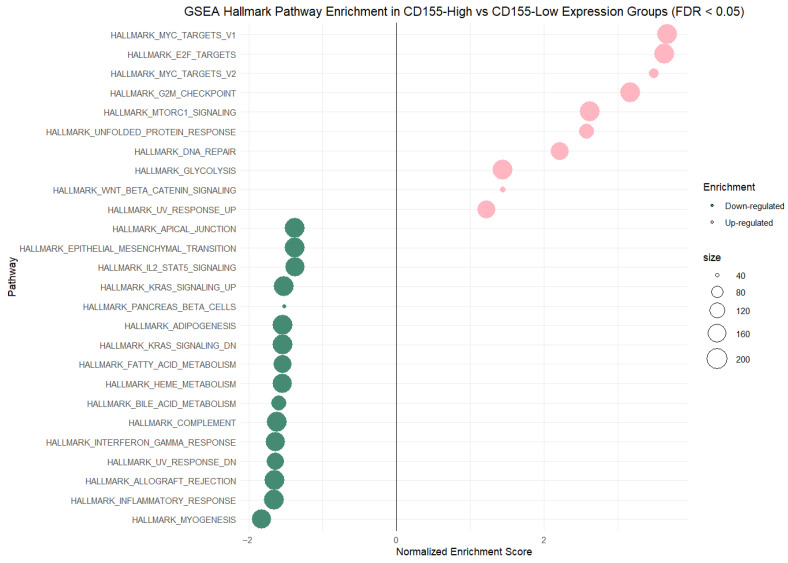
Gene set enrichment analysis (GSEA) of Hallmark pathways comparing high CD155 gene expression CRC tumor group versus low CD155 expression CRC tumor group (FDR < 0.05). Dots represent normalized enrichment scores (NESs) for each pathway, with point size proportional to gene set size.

On the other hand, GSEA comparing CD155-high vs. CD155-low samples (FDR < 0.05) revealed 26 unique Hallmark pathways, including 10 pathways enriched in the CD155-high group (NES > 0) and 16 pathways were enriched in the CD155-low group (NES < 0). The CD155-high expression group showed strong enrichment of cell cycle and proliferative signatures, led by Hallmark MYC Targets V1 (NES = 3.67), Hallmark E2F Targets (NES = 3.63), Hallmark MYC Targets V2 (NES = 3.48), and Hallmark G2M Checkpoint (NES = 3.17). Also, stress/metabolic-related pathways have been significantly enriched in this group, including Hallmark Unfolded Protein Response (NES = 2.58), Hallmark DNA Repair (NES = 2.21), Hallmark Glycolysis (NES = 1.44), and Hallmark WNT Beta Catenin Signaling (NES = 1.44). In contrast, the CD155-low group was enriched for immune/inflammatory and related programs, including Hallmark Inflammatory Response (NES = −1.65), Hallmark Interferon Gamma Response (NES = −1.64), Hallmark Allograft Rejection (NES = −1.64), and Hallmark Complement (NES = −1.62), as well as tissue/other signatures such as Hallmark Myogensis (NES = −1.83). Additional negatively enriched pathways included lipid/porphyrin-associated metabolism, such as Hallmark Bile Acid Metabolism (NES = −1.59), Hallmark Fatty Acid Metabolism (NES = −1.55), among others, indicating that CD155 stratification separates a predominantly proliferative, MYC/E2F-driven state from an immune/inflammation-associated state in the examined dataset. Figure 12 demonstrates the GSEA results for CD155 gene expression, and detailed statistical outcomes of GSEA for high versus low CD155 expression are presented in Table S7.

To summarize, GSEA indicates that, in CRC, TIGIT primarily marks an immune-inflamed program, with the highest enrichment clustered around interferon signaling (IFN-g/IFN-a) and broader inflammatory cytokine axes (TNFa/NF-kB, IL6-JAK-STAT3), while the most depleted signatures group into tumor-intrinsic growth/metabolic programs (notably MYC targets and oxidative phosphorylation). In contrast, CD155 stratifies tumors mainly by proliferative/cell cycle activity, showing the strongest enrichment for MYC- and E2F-driven transcriptional programs and G2M checkpoint, whereas the lowest enrichment clusters into immune/inflammatory modules and selected differentiation-related pathways. Taken together, these profiles suggest a clear dissociation: TIGIT-high CRC aligns with immune activation and inflammatory signaling, whereas CD155-high CRC aligns with proliferative, oncogenic cell cycle circuitry accompanied by relative suppression of immune-response pathways, highlighting biologically distinct contexts for the TIGIT/CD155 axis in CRC.

## 3. Discussion

TIGIT was identified in 2009 as a coinhibitory receptor that serves as a key modulator of both adaptive and innate immune responses via diverse molecular mechanisms. It is expressed on selected NK cell subsets and B cells, particularly those with regulatory properties, as well as on various T cell subsets, such as regulatory T cells (Tregs), Tr1 cells, T follicular helper (Tfh) cells, and exhausted or dysfunctional CD8^+^ T cells. Moreover, TIGIT expression has also been reported on various innate-like lymphocyte populations, such as mucosa-associated invariant T (MAIT) cells, invariant natural killer T (iNKT) cells, innate lymphoid cells (ILCs), and macrophages, where its engagement can contribute to amplified immunosuppressive responses within the TME. TIGIT interacts with several nectin and nectin-like ligands, most prominently CD155 (PVR), and also CD112 and CD113. Ligation of TIGIT on dendritic cells (DCs) induces CD155 phosphorylation and triggers downstream MAPK/ERK signaling, promoting the development of immunoregulatory DCs marked by diminished IL-12 production and elevated IL-10 levels, consequently impairing T cell and NK cell activity. In addition, binding of TIGIT to CD155 inhibits IFN-g secretion through β-arrestin-2-dependent suppression of NF-κB signaling and reduces the cytotoxic capacity of NK cells. Elevated expression of TIGIT and CD155 has been demonstrated in multiple human cancers, including melanoma [37], lung adenocarcinoma [38], hepatocellular carcinoma [39], pancreatic cancer [40], and gastric cancer [12], among other types of cancer, supporting the relevance of this immune axis as a therapeutic target in oncology.

In CRC, increased expression of TIGIT and CD155 has also been reported, and several studies have suggested a potential prognostic relevance of these molecules. However, the available data on their prognostic significance are still evolving and are not entirely consistent. Kitsou et al. observed that high TIGIT expression tended to be associated with improved overall survival (OS) [41]. Similarly, in the study by Meyiah et al., which examined combined expression patterns of multiple immune checkpoints rather than single markers, higher frequencies of PD-1^+^TIGIT^+^ and PD-1^+^TIGIT^+^ CD8^+^ tumor-infiltrating lymphocytes (TILs) were significantly correlated with longer disease-free survival (DFS) compared with lower frequencies of these subsets. In the same work, the authors validated their findings by analyzing disease-free survival (DFS) and OS according to TIGIT gene expression levels in bulk tumors from the TCGA CRC cohort, showing that patients with high TIGIT expression had better DFS and OS [42]. By contrast, numerous other studies have linked TIGIT overexpression to unfavorable prognosis and more advanced disease. Cao et al., analyzing transcriptomic data from CRC patients, reported that elevated TIGIT expression was associated with poorer OS and DFS [43]. In line with this, Shao et al. demonstrated that increased frequencies of CD3^+^TIGIT^+^ T cells in both peripheral blood and tumor tissue of CRC patients were associated with worse clinical outcome, and that these cells were more abundant than in healthy donors [44]. Consistently, Liang and Zhu et al. reported higher TIGIT expression in 139 tumor samples compared with 68 normal tissues, along with increased proportions of TIGIT^+^CD3^+^, TIGIT^+^CD4^+^, TIGIT^+^CD8^+^ T cells, and elevated TIGIT expression in NK cells within tumor tissue relative to normal mucosa. Moreover, TIGIT expression was further increased in lymph node and liver metastases compared with primary tumors [45]. Murakami et al. similarly found elevated levels of both TIGIT and CD155 in cancerous versus normal tissue. In their study, the proportions of TIGIT^+^CD3^+^, TIGIT^+^CD4^+^, and TIGIT^+^CD8^+^ T cells in the peripheral blood of CRC patients were higher than in healthy donors, and these subsets were even more enriched in tumor tissue than in matched blood samples. TIGIT^+^CD3^+^ T cell infiltration was also greater in tumor tissue than in adjacent normal mucosa, and TIGIT overexpression correlated with tumor progression and adverse prognosis [46]. In dMMR CRC, both TIGIT protein and mRNA levels were higher in tumor tissue than in adjacent mucosa and were associated with a higher TNM stage [47]. CD155 expression was shown to be upregulated in tumor-associated macrophages (TAMs) derived from CRC tissue compared with adjacent non-malignant tissue, and this increase was linked to advanced tumor stage and reduced OS [48]. Likewise, Liang and Liu et al. reported elevated CD155 expression at both the mRNA and protein levels, with higher expression correlating with poorer prognosis [49].

The apparent discrepancies between studies reporting either favorable or unfavorable associations of high TIGIT or CD155 expression with clinical outcome in CRC are likely multifactorial. First, TIGIT and CD155 are expressed on phenotypically and functionally diverse cell populations, including tumor cells, tumor-associated macrophages, NK cells, and multiple T cell subsets. Their presence in the TME may be associated with an inflammatory antitumor response, which may result in improved DFS and OS. In contrast, when TIGIT and/or CD155 upregulation predominates on immunosuppressive cell types—such as Tregs or TAMs—or on tumor cells themselves, it may reflect a more advanced state of immune escape and thus associate with poor prognosis. Considerable methodological heterogeneity likely further contributes to these divergent results, as studies differ in analytical methods, such as bulk transcriptomics, IHC, flow cytometry, or ELISA. Tumor-intrinsic features such as MSI status, mutational burden, co-expression of other immune checkpoints, and differences in treatment exposure may also modulate the biological and clinical significance of the TIGIT/CD155 axis. Together, these considerations suggest that the prognostic value of TIGIT and CD155 may be highly context dependent.

In the present study, we observed a significantly higher expression of both TIGIT and CD155 proteins in CRC tissues compared with matched non-malignant surgical margin samples. The clinical distribution of the patients was similar to that described previously for this cohort, with most cases classified as intermediate stages of disease (stage II and III: 25.19% and 45.80%, respectively), and thus broadly representative of the general CRC population. In our cohort, TIGIT and CD155 protein levels were quantified in tissue homogenates using an ELISA-based approach, providing a measure of their overall abundance within the tumor compartment rather than cell-type-specific or spatially resolved expression. In this setting, we did not detect any significant associations between TIGIT or CD155 concentrations and TNM classification, clinical stage, or histological grade. These findings indicate that, at the level of whole-tissue protein content, upregulation of the TIGIT/CD155 axis appears to be a relatively common feature of CRC that does not segregate with conventional indicators of tumor stage. Methodologically, the use of tissue homogenates integrates signals from tumor cells, stromal elements, and multiple immune cell subsets, which may mask opposing effects exerted by distinct cellular compartments. Thus, the absence of correlations with examined clinicopathological parameters in our study is in line with the notion that the clinical relevance of TIGIT and CD155 likely depends on their distribution across specific immune and tumor cell subsets.

MMR is a highly conserved cellular process that involves a set of proteins responsible for the recognition and subsequent repair of mismatched bases, which arise during DNA replication, genetic recombination, or as a consequence of chemical or physical DNA damage [50]. MSI is a hypermutable phenotype that results from the loss of DNA mismatch repair activity [51,52]. CRC can be broadly categorized into two discrete groups on the basis of mutation patterns: tumors with deficient mismatch repair (dMMR) or a microsatellite instability-high (MSI-H) signature and a high overall mutation burden, and tumors with proficient mismatch repair (pMMR) or microsatellite-stable (MSS) status and a much lower mutation burden [53]. CRCs with MSI-H status demonstrate divergent clinicopathological characteristics, particularly a predilection for the proximal colon, mucinous phenotype, poor differentiation, lower frequencies of KRAS and TP53 mutations, increased immune cell infiltrates, and a reduced rate of metastasis. The majority (~85%) of CRCs are pMMR or MSS and derive little or no benefit from immune checkpoint inhibitors (ICIs) monotherapy. The remaining (~15%) of CRCs exhibit MSI, which is characterized by elevated rates of small insertion-deletion mutations and point mutations within microsatellites [23,54]. In sporadic CRC, MSI is predominantly caused by epigenetic silencing of MLH1 due to promoter hypermethylation [55].

Regionalized promoter hypermethylation is a common feature of sporadic MSI-H CRC and is closely associated with the CpG island methylator phenotype (CIMP) [52,56]. Hypermethylation of CpG islands (CpGIs) plays a major role in gene inactivation within the TME [57]. Nair et al. performed a study to analyze whether the promoter methylation profiles of selected immune checkpoint genes, including TIGIT, differ significantly between CRC tumor and non-tumor colorectal tissues [58]. They demonstrated that the mean demethylation percentage of the TIGIT promoter in CRC tumor tissue was significantly higher than in non-tumor tissue. Moreover, the abundance of the repressive histone mark H3K9me3 in the promoter regions of TIGIT was significantly lower in CRC tumors compared with non-tumor tissues. These findings indicate that transcriptional upregulation driven by promoter demethylation, together with a reduced abundance of repressive histone marks in the TIGIT promoter region, may play an important role in regulating TIGIT expression in the CRC TME [58]. Although CIMP-high is defined on the basis of focal, high-level hypermethylation of CpG islands, these tumors, like other cancers, also display elements of global hypomethylation in selected genomic regions [57,59]. Such a disturbed epigenetic landscape may favor both the silencing of tumor suppressor genes and the derepression of immune-regulatory genes, including TIGIT. In line with the findings of Nair et al., upregulation of TIGIT expression may, therefore, occur via this epigenetic mechanism. Although Nair et al. did not analyze MSI-H and MSS tumors separately, their data support a model of epigenetic derepression of TIGIT that, in the epigenetically unstable context of MSI-H tumors, might be expected to occur more frequently [58]. By contrast, Kitsou et al. demonstrated that increased TIGIT expression correlated significantly with tumor-infiltrating lymphocyte (TIL) load, whereas no association was found between high mutational burden and dysregulated TIGIT expression [41].

Only a limited number of studies have examined the association between TIGIT expression and direct MSI/dMMR status. Zhou et al. analyzed a cohort of 60 dMMR colorectal tumors and showed that TIGIT expression was increased on regulatory T cells (Tregs), but remained low on activated T cells, when compared with normal colonic mucosa [47]. They also reported higher TIGIT mRNA levels in dMMR tumors relative to matched non-neoplastic tissue. However, this study did not compare TIGIT expression between dMMR/MSI and pMMR/MSS tumors; the analyses were limited to dMMR tumors and normal mucosa as the control group [47]. Using a different approach, Wen et al. analyzed TCGA data from 33 cancer types and demonstrated that, in CRC, TIGIT expression correlated positively with MSI-related genes and with tumor mutational burden [60]. Subsequently, Zaravinos et al. based conducted a TCGA analysis, found that high TIGIT expression correlated significantly with increased immune cytolytic activity (CYT) in CRC [61]. CYT-high tumors exhibited elevated TIGIT expression in both colon adenocarcinoma (COAD) and rectal adenocarcinoma (READ) datasets. TCGA-based analyses from the same study also showed that MSI CRCs have higher expression of several immune checkpoint molecules, including TIGIT, compared with MSS tumors. Zaravinos et al. also demonstrated that CYT is associated with distinct mutational profiles characteristic of CRC, with mutation load being significantly higher in MSI tumors. They also observed that high CYT was significantly associated with BRAF mutations, while no such association was found for KRAS or PIK3CA mutations, which were also evaluated in our study. In addition to these in silico findings, the authors assessed TIGIT expression by RT-qPCR in a cohort of 72 CRC tumors, comprising 12 MSI-H, 13 MSI-L and 47 MSS cases, and demonstrated significantly higher TIGIT expression in MSI-H tumors than in both MSI-L and MSS groups [61]. The association between CD155 expression and MSI status has been less reported in current data. Zhang et al., using TCGA data, reported negative correlations between CD155 expression and both tumor mutational burden and MSI in CRC [62].

In line with the above findings, our results further support that MSI status is associated with enhanced immune checkpoint expression in CRC. In our cohort, tumors classified as MSI on the basis of IHC assessment of MMR protein loss showed significantly higher TIGIT protein levels compared with the MSS tumor group. These data are consistent with transcriptomic observations from the analyses by Wen et al. and Zaravinos et al., who reported positive associations between TIGIT expression, MSI-related signatures, tumor mutational burden, and immune cytolytic activity in CRC [34,35]. Together with the epigenetic data of Nair et al. showing promoter demethylation and reduced H3K9me3 at the TIGIT locus in CRC tissue [30], our findings support a model in which MSI-driven genomic instability and deregulated epigenetic landscape act in concert with a highly inflamed TME to promote upregulation of TIGIT and other immune checkpoint molecules in MSI CRC. By contrast, we did not observe significant differences in CD155 protein concentrations between MSI and MSS tumors, despite the weak negative correlations between CD155 expression, tumor mutational burden, and MSI reported at the transcriptomic level by Zhang et al. [36]. This discrepancy may reflect differences between mRNA and protein regulation of CD155. Overall, our data suggest that, within the TIGIT/CD155 axis, TIGIT expression is associated with MSI status at the protein level in CRC, reinforcing the concept that MSI-associated epigenetic and immunological alterations preferentially favor the derepression and induction of immune checkpoint receptors such as TIGIT.

In our study, we observed increased TIGIT protein levels in *BRAF*-mutated tumor group compared with the *BRAF* wild-type CRC tumor group, whereas no similar association was found for CD155 concentrations. To the best of our knowledge, no previous experimental work has evaluated the associations between *BRAF* mutational status and TIGIT/CD155 expression directly. Nevertheless, the influence of *BRAF* mutations on the immune TME in CRC is well documented. *BRAF* mutations occur in approximately 10% of CRC cases, with over 90% corresponding to the *BRAF V600E* variant, and are frequently associated with poor differentiation, advanced T stage, and an elevated risk of metastasis [30,31,63]. Functionally, oncogenic *BRAF* drives persistent activation of the MAPK/ERK pathway, which attenuates upstream EGFR/RAS signaling and thereby perturbs antitumor immune surveillance at multiple stages of the cancer-immunity mechanisms [32,33,64]. Moreover, *BRAF* mutations often co-occur with MSI status in CRC and, according to the consensus molecular subtype (CMS) classification, define the MSI-immune CMS1 subtype, which is characterized by pronounced immune cell infiltration and activation [17]. In this context, the elevated expression of immune checkpoint molecules such as TIGIT in *BRAF*-mutated tumors is not unexpected. Our findings are thus in line with established pathogenetic evidence in *BRAF*-mutated, MSI-immune CRC and reinforce the concept of TIGIT as a relevant immunoregulatory checkpoint that may constitute a particularly promising therapeutic target within this molecular subset of CRC.

*KRAS* is mutated in approximately 35–45% of sporadic CRC cases and is associated with an unfavorable prognosis [25,27,28,65]. *KRAS* mutations are linked to dysregulation of the EGFR and MAPK signaling pathways, which are critically involved in the control of cell proliferation, differentiation, apoptosis, and senescence [27]. Beyond these well-established oncogenic functions, *KRAS* mutation may also exert an impact on the immunological landscape of the CRC TME. According to Lal et al., *KRAS* mutation in CRC is associated with a globally more immunosuppressed TME, characterized by attenuation of Th1/cytotoxic immunity. *KRAS*-mutant tumors have reduced expression of a Th1-centric co-ordinate immune response cluster (CIRC), with particularly lower infiltration of cytotoxic T cells, Th1 cells, and neutrophils, and broad downregulation of multiple immune-related Hallmark pathways, most notably the interferon-g pathway [66]. Moreover, according to a study by Edin et al., *KRAS* mutation in CRC is consistently associated with a less inflamed, more immunosuppressed TME. Using the IHC method on tumor front and center, the authors show that *KRAS*-mutated tumors have significantly lower infiltration of CD8^+^ cytotoxic T cells and T-bet^+^ Th1 cells compared with *KRAS* wild-type cases, particularly at the tumor front, where immune-tumor interactions are most critical. In the tumor center, similar but weaker patterns are seen, and *KRAS* mutation is additionally linked to increased regulatory T cell (FoxP3^+^) infiltration, further supporting an immunosuppressive phenotype [67]. *KRAS*-driven tumors also show broader metabolic and signaling changes (including dysregulated glucose/glutamine/fatty acid metabolism and activation of MAPK and HIF-1-related cascades) that support tumor growth and indirectly reinforce immune escape [29]. Taken together, the available data indicate that oncogenic *KRAS* does not simply reduce the quantity of TILs but qualitatively remodels the immune landscape in a way that is highly relevant for immune checkpoint regulation. When integrating these data with our own results, it is important to note that, despite the strong mechanistic basics for *KRAS*-driven immune remodeling, we did not observe significant differences in TIGIT or CD155 protein levels between *KRAS*-mutant and *KRAS*-wild-type tumors in our cohort, as measured by ELISA in tissue homogenates. In our study, we have observed significantly higher CD155 concentration in *KRAS* codon 146 mutated tumors. However, given the limited number of cases harboring the *KRAS* codon 146 mutation in our cohort, the observed association with CD155 should be considered exploratory, and it warrants validation in larger, independent cohort. This apparent discrepancy suggests that the impact of *KRAS* on immune checkpoint regulation may be more qualitative and compartment-specific than reflected by total tissue concentrations. In particular, *KRAS* mutation has been linked to shifts in the composition and functional state of tumor-infiltrating immune cells rather than to a uniform increase or decrease in global TIGIT/CD155 expression across the entire tissue. These observations underscore the need for cell type-resolved and spatially approaches, to more precisely dissect how *KRAS* mutations reshape TIGIT-dependent regulatory molecules within the CRC TME.

TIGIT^+^CD8^+^ T cells have been shown to secrete lower levels of IFN-g, IL-2, and TNF compared to TIGIT^−^CD8^+^ T cells, highlighting how TIGIT can impair CD8^+^ T cell function. Furthermore, TIGIT was found to modulate the expression of receptors such as TIGIT, LAG-3, and PD-1 on CD8^+^ T cells, contributing to T cell exhaustion [45]. As reported by Li et al., blocking TIGIT increases the percentage of cytokine-secreting CD8^+^ T cells and activates the NF-κB pathway [68]. Huang et al. demonstrated the role of TIGIT and CD155 in regulating CD8^+^ T cell glucose metabolism via the PI3K/AKT/mTOR pathway, resulting in subsequent modifications in the TME and cytokine production [69]. TIGIT has also been shown to play a crucial role in other types of cancer. In gastric cancer, CD8^+^ T cells expressing the TIGIT receptor were involved in regulating a number of different processes within the TME, including proliferation, metabolism, and cytokine secretion. TIGIT signaling reduced AKT/mTOR pathway activation through decreased phosphorylation [12]. In castration-resistant prostate cancer (CRPC), TIGIT inhibition increased NK cell-mediated chemotaxis of T cells, improved cytotoxicity against CRPC cells, and cytokine production. Additionally, the MEK/ERK and NF-κB signaling pathways were shown to be involved in these changes [70]. CD155 can regulate cell motility and proliferation in mouse embryonic fibroblasts. It increases platelet-derived growth factor (PDGF)-mediated Ras-Raf-MEK-ERK activation by acting downstream of the PDGF receptor and upstream of Ras [71]. In ESCA, CD155 expression was found to be correlated with PD-1 and PD-L1. Furthermore, CD155 was associated with cell proliferation, and its downregulation led to decreased proliferation by disrupting the cell cycle and inducing apoptosis, supposedly through the inhibition of the PI3K/Akt and MAPK signaling pathways [72]. CD155 was suggested to be involved in angiogenesis. It may regulate VEGF-meditated angiogenesis through modulation of the interaction between VEGFR2 and integrin avβ3. It was reported to be involved in regulation of the VEGFR2-mediated Rap1-Akt signaling pathway [73]. Briukhovetska et al. reported that IL-22 can induce the expression of CD155, which binds to CD226, activating NK cells. However, excessive activation led to decreased CD226 expression, altered NK cell activity, and reduced IFN-g secretion, ultimately increasing the metastatic properties of lung cancer cells [74].

Integration of our own cytokine correlation analysis and GSEA results suggests that the TIGIT/CD155 immune checkpoint axis may be associated with multiple immune-related pathways in CRC tumor tissues. In our protein measurements of selected 48 cytokines, chemokines, and growth factors, only a few significant correlations were detected, but IFN-g distinguish as a shared correlate, showing positive associations with TIGIT and CD155. CD155 was also positively correlated with TRAIL, IL-1Ra, M-CSF, and PDGF-bb, suggesting that higher CD155 can accompany a broader inflammatory or growth-factor tumor milieu. These experimental findings fit well with the indicated GSEA results: TIGIT-high samples were enriched for immune and inflammation-related pathways, including the IFN-g response, consistent with TIGIT marking a more immune-inflamed TME in tumor tissues. In contrast, CD155-high samples were enriched mainly for proliferation and growth programs (MYC/E2F/G2M signatures), while immune/inflammatory pathways (including IFN-related programs) were more prominent in CD155-low, producing an apparent opposite pattern between TIGIT and CD155 at the pathway level. Importantly, this does not conflict with previous functional studies showing that TIGIT/CD155 signaling can reduce IFN-g production by T and NK cell [45,74]. In bulk CRC tumors, positive correlations can still occur because immune-inflamed samples often show both higher IFN-g and higher checkpoint molecules, reflecting immune activation together with feedback inhibition, which means that higher immune checkpoint expression may reduce further immune activation and cytokine production in consequence. Moreover, integrating our experimental PCA/correlation results with the GSEA findings places TIGIT in CRC firmly in the context of an IFN-g-driven, immune-inflamed TME, where active antitumor immunity coexists with checkpoint-mediated restraint. As an inhibitory receptor on T and NK cells, TIGIT is plausibly induced as part of adaptive immune resistance: sustained IFN-g signaling and downstream IFN-g–dependent cytokine programs reflect immune engagement, while concomitant TIGIT upregulation may potentially represent a compensatory inhibition that can limit cytotoxic effector function and promote functional exhaustion. Translationally, this co-occurrence suggests that TIGIT-high/IFN-g-high CRC tumors may define a clinically actionable subgroup characterized by pre-existing immune activation but incomplete tumor rejection, in which a therapeutic strategy based on inhibitory signaling could be potentially beneficial. In contrast, CD155 showed a broader association pattern that included growth-factor and myeloid/stromal mediators, such as PDGF-bb, M-CSF, and TRAIL and, at the pathway level, preferential enrichment of proliferative programs (MYC/E2F/G2M), suggesting that CD155 abundance in bulk tissue may more strongly reflect tumor/stromal remodeling and tumor cell–intrinsic programs than immune activation alone. Importantly, this divergence implies that interpreting the TIGIT/CD155 axis and prioritizing TIGIT-directed immunotherapy strategies may require patient stratification by immune context (MSI/BRAF-associated immune-inflamed subsets) because TIGIT-high tumors could represent settings where combination checkpoint blockade is biologically more plausible, whereas CD155-high tumors may indicate a proliferative, microenvironmentally remodeled state that could benefit from different or additional combinatorial approaches. The precise associations between the TIGIT/CD155 axis proteins and interferon-related signaling in CRC require further investigation. Future studies using approaches that resolve cell-type specificity and enable functional testing will be needed to determine whether these associations reflect a direct regulatory link or are driven primarily by differences in CRC TME composition.

The TIGIT/CD155 axis is considered as a potential therapeutic target in many solid tumors. In Head and Neck Squamous Cell Carcinoma (HNSCC), both in vitro and in vivo blockade of TIGIT increased antitumor immune responses. In vitro studies showed decreased immunosuppressive ability of myeloid-derived suppressor cells (MDSC), supposedly achieved by decreased expression of Arg1, and Tregs by decreased TGFβ1 secretion. In vivo studies showed that blockade of TIGIT results in activation of CD8^+^ T cells, increasing their effector function and lowering number of Tregs [75]. In an ovarian cancer (OC) mouse model, TIGIT function was blocked using Anti-TIGIT monoclonal antibodies. This resulted in decreased proportion of CD4^+^ Tregs, while no effect was observed on CD4^+^ and CD8^+^ T cells or NK cells. TIGIT inhibition diminished the immunosuppressive activity of splenic CD4^+^ Tregs. Moreover, anti-TIGIT treatment improved the survival rate of OC mice [76]. Johnston et al. reported that blockade of both TIGIT and PDL1 in CT26 tumor-bearing mice significantly reduced tumor growth and led to complete responses in most mice. Similar results were observed in other tumor models [77]. First-in-human phase 1 study of anti-TIGIT antibody vibostolimab plus pembrolizumab was well tolerated in advanced solid tumors and demonstrated antitumor activity [78]. In the CITYSCAPE study, 135 patients with chemotherapy-naive, PD-L1-positive, recurrent or metastatic NSCLC received tiragolumab plus atezolizumab or placebo plus atezolizumab. It was observed that the group of tiragolumab plus atezolizumab had higher response rates and progression-free survival in comparison to placebo plus atezolizumab. The combination was tolerated, with a safety profile similar to atezolizumab in monotherapy, supporting its potential as a promising treatment option [79]. Overall, anti-TIGIT antibodies show limited efficacy in terms of monotherapy. However, its combination with anti-PD-(L)1 shows better results. Lack of biomarkers, which could predict efficacy of such treatment strategy, limits possible optimization of such immune therapies [80,81]. In terms of CD155 inhibition, there are limited studies. However, recombinant nonpathogenic polio-rhinovirus chimera (PVSRIPO), which targets CD155, was shown to be a possible therapeutic option in GBM [82].

Our findings have potential implications for TIGIT/CD155-directed immunotherapy in CRC, particularly in molecularly defined subgroups. We observed higher TIGIT protein levels in MSI tumors and in *BRAF*-mutant CRC, suggesting that TIGIT upregulation may mark an immune-inflamed context in which checkpoint blockade strategies could be especially relevant. In line with this, our cytokine analysis identified IFN-g as a shared positive associate of both TIGIT and CD155, which is consistent with the idea that immune activation and compensatory checkpoint upregulation can co-occur in tumor tissue. Given that anti-TIGIT monotherapy has generally shown limited efficacy, while combinations with anti-PD-(L)1 perform better in several settings, our data support the rationale for exploring TIGIT blockade as an add-on strategy, potentially with greatest value in MSI and/or *BRAF*-mutant CRC, where TIGIT/CD155 expression could be significantly upregulated.

Despite the results we observed in this study, there are significant limitations of this study, stemming from the methods used, that should be noted. An important limitation of this study is that TIGIT and CD155 were quantified in whole-tissue homogenates, which integrate signals from tumor cells, stromal elements, and multiple immune cell subsets. As a result, this approach cannot resolve the cellular source or spatial localization of the measured proteins: whether CD155 derives predominantly from malignant epithelial cells, cancer-associated fibroblasts/endothelium, or tumor-associated macrophages, and whether TIGIT reflects exhausted CD8^+^ T cells, Tregs, or other lymphocyte compartments. It may, therefore, mask opposing effects occurring in distinct TME niches. A second key limitation is that we assessed only TIGIT and CD155 without parallel evaluation of CD226 (DNAM-1) and CD96 (TACTILE), which compete for CD155 binding and can deliver signals with different (activating vs. inhibitory and context-dependent) functional consequences. Accordingly, we could not quantify the counterbalance within the CD155-(TIGIT/CD226/CD96) receptor network that ultimately shapes immune responses. The lack of in situ analyses, such as IHC/IF or cell-based profiling, further limits interpretation by precluding direct attribution of expression patterns to specific compartments and by not accounting for CRC tumors’ heterogeneity. For instance, CD155 expression may arise from non-mutant tumor subclones or from non-malignant stromal cells, which complicates mutation-expression associations in bulk tissue. Finally, our conclusions are based on cross-sectional measurements and correlations without functional validation of how the TIGIT/CD155 axis affects effector activity in CRC, and differences between mRNA and protein regulation, as well as pre-analytical factors (tissue composition, variable stromal content, and protein stability) may influence protein concentrations measured in homogenates.

## 4. Materials and Methods

### 4.1. Study Group Characteristics

A total of 262 tissue samples were collected and analyzed, including 131 CRC tissue samples in the study group and 131 surgical margin tissue samples in the control group. The present study was performed in the same CRC patient cohort as reported previously [83].

The surgical margin tissue samples were evaluated to exclude any pathological signs of neoplasia. Eligibility criteria comprised (1) provision of written informed consent, (2) age ≥ 18 years, and (3) histopathological verification of colorectal adenocarcinoma and/or confirmation of tumor-free resection margins. Individuals failing to satisfy these requirements were not enrolled in the study. The study protocol was approved by the Research Ethics Committee (PCN/0022/KB1/42/VI/14/16/18/19/20). The cohort had a median age of 65 years (±9.22). Most tumors were left-sided (distal), accounting for 71.76% of cases. Stage III disease predominated (45.80%), followed by stage II (25.19%), while stages I and IV were equally represented (14.50% each). A detailed summary of clinicopathological characteristics is provided in Table 1.

### 4.2. TIGIT and CD155 Protein Concentration Evaluation by ELISA

A total of 131 CRC tumors and 131 margin tissues were homogenized according to a previously established protocol [84]. Samples were homogenized in phosphate-buffered solution using a PRO 200 homogenizer (PRO Scientific Inc., Oxford, CT, USA) at 10,000 rpm and further disrupted using ultrasonic sonication (UP100, Hilscher, Hattersheim am Main, Germany). Total protein concentration was determined using the pyrogallol red assay (Sentinel Diagnostics, Milan, Italy) on a Technicon RA-XTTM analyzer (Technicon Instruments Corporation, Mahopac, NY, USA) at 600 nm and 37 °C. TIGIT and CD155 levels were measured with commercial ELISA kits (SEN056Hu and SEB550Hu; Cloud Clone, Wuhan, China) according to the manufacturers’ instructions, with sample dilutions optimized in pilot experiments. The assay detection limits were <0.114 ng/mL for TIGIT and <0.058 ng/mL for CD155. Protein concentrations were normalized to total protein content and reported as ng per mL of total protein.

### 4.3. Assessment of Mutational Profile of CRC Tumors

The mutational landscape of *KRAS*, *NRAS*, *PIK3CA*, *AKT1*, and *BRAF* was interrogated by real-time PCR, following methods described in our earlier work [85]. Genomic DNA was extracted from fresh-frozen CRC tumor specimens stored at −80 °C using an automated isolation platform and the Mag-Bind Blood & Tissue DNA HDQ 96 Kit (M6399-00) (Omega Bio-tek Inc., Norcross, GA, USA), following the manufacturer’s instructions. DNA levels were quantified spectrophotometrically, and all isolates were adjusted to 2 ng/μL in accordance with the RT-PCR assay requirements. Mutation testing was then conducted with the CRC-RT48 Mutation Detection Panel for real-time PCR (EntroGen, Woodland Hills, CA, USA) using the QuantStudio™ 5 Real-Time PCR System (Thermo Fisher Scientific, Waltham, MA, US) for amplification and signal acquisition. The panel interrogates hotspot regions in *KRAS* (exons 2–4), *NRAS* (exons 2–4), *PIK3CA* (exons 9 and 20), *AKT1* (exon 4), and *BRAF* (exon 15), enabling detection of recurrent variants including *KRAS* (codons 12/13, 59, 61, 117, and 146), *NRAS* (codons 12/13, 59, 61, 117, and 146), *PIK3CA* (codons 542/545 and 1047), *AKT1 E17K*, and *BRAF V600* (Table 7).

### 4.4. Microsatellite Instability (MSI) Evaluation

MSI status was assessed by immunohistochemistry (IHC) on formalin-fixed, paraffin-embedded (FFPE) CRC tissue blocks according to previously reported procedures [86,87]. In total, MSI evaluation was performed in 79 tumor samples. Sections (4 μm) were cut and stained using a Dako Autostainer Link 48. After deparaffinization and rehydration, antigen retrieval was carried out with EnVision Flex Target Retrieval Solution (High pH; Dako, Carpinteria, CA, USA) at 95 °C for 20 min. Following a blocking step, slides were incubated with monoclonal antibodies against the mismatch repair proteins MSH2 (clone G219-1129, Cell Marque; 1:400, 30 min), MSH6 (clone 44, Cell Marque; 1:100, 45 min), PMS2 (clone MRQ-28, Cell Marque; 1:50, 40 min), and MLH1 (clone G168-728, Cell Marque; 1:100, 40 min). Signal detection employed the EnVision FLEX HRP system (Dako) with DAB (3,3′-diaminobenzidine) as chromogen, followed by hematoxylin counterstaining and coverslipping. MMR protein expression was interpreted based on nuclear staining in tumor cells, with stromal and inflammatory cells used as internal positive controls. Tumors were considered as MSI-positive when complete loss of nuclear staining was observed for any of the following markers: MSH2/MSH6, isolated PMS2, MLH1/PMS2, or isolated MSH6.

### 4.5. Principal Component Analysis (PCA) on Cytokine, Chemokine, and Growth Factor Concentration in CRC Homogenates

The concentrations of cytokines, chemokines, and growth factors in homogenized tissue samples from 77 colorectal cancer (CRC) cases were measured using the Bio-Plex Pro Human Cytokine Screening Panel, 48-Plex (Bio-Rad Laboratories, Hercules, CA, US), in accordance with the manufacturer’s manual. The obtained concentration data were normalized to the total protein level in CRC tissue homogenates and subsequently categorized into functional groups based on Gene Ontology (GO) classifications, KEGG pathway, and WikiPathways annotations (Table 8) [88,89,90]. We performed a principal component analysis (PCA) on base-10 log–scaled, normalized expression measurements to summarize the major sources of variation in the dataset. The eigen decomposition was computed from the variance–covariance structure, and the first three components were retained for subsequent analyses. To obtain a clearer and more readily interpretable loading pattern, we applied a varimax rotation. Component scores were then related to TIGIT and CD155 protein levels using Spearman’s rho, and associations were considered significant at *p* < 0.05. All analyses were conducted in R (RStudio v4.4.1) using the factoextra package.

### 4.6. Gene Set Enrichment Analysis (GSEA) for TIGIT and CD155 Gene Expression on CRC Data

Gene set enrichment analysis (GSEA) was performed in R (RStudio v 4.4.1) using the “FieldEffect-Crc” resource, focusing on colorectal cancer (CRC) specimens from Cohort A [24]. The dataset contains Salmon-derived transcript-level abundance estimates that were aggregated to gene-level expression with tximport, and comprises 834 colorectal tissue samples spanning tumor, matched adjacent normal, and healthy tissues [91]. Data were accessed via ExperimentHub and restricted to tumor-derived CRC samples (*n* = 311). Differential expression was computed with DESeq2 (DESeq), which models count data and accounts for differences in library size; normalized counts were extracted for downstream analyses. Samples were then stratified by the median expression of TIGIT (ENSG00000181847) and CD155/PVR (ENSG00000073008), classifying cases above the median as high expression (*n* = 156) and those below as low expression (*n* = 155). ENSEMBL identifiers were converted to gene symbols using org.Hs.eg.db, and a ranked gene list derived from the differential expression statistics was used as input for GSEA. Enrichment testing was carried out with MSigDB Hallmark gene sets [92,93], using the fgsea algorithm, employing 10,000 permutations and restricting analysis to gene sets comprising 15–400 genes. Pathways with FDR-adjusted *p*-values < 0.05 were retained and reported after ranking by normalized enrichment score (NES).

### 4.7. Statistical Analyses

Data distribution was evaluated with the Shapiro–Wilk test, and protein concentrations were log10-transformed to improve comparability across samples. Paired differences in TIGIT and CD155 levels between CRC tumor tissue and matched surgical margins were first examined using the non-parametric Wilcoxon signed-rank test. To further account for between-patient heterogeneity and the paired structure of the dataset, linear mixed-effects models were then fitted with patient ID as a random intercept and tissue type (tumor vs. margin) as a fixed effect (Protein ~ TissueType + (1|PatientID)) using restricted maximum likelihood (REML). Fixed-effect degrees of freedom were estimated with Satterthwaite’s approximation, and corresponding *p*-values were reported. Model diagnostics included visual inspection of residuals to assess homoscedasticity and approximate normality. Associations between protein levels and TNM variables were assessed using Kendall’s tau. Differences in protein expression according to mutational status were tested with the Mann–Whitney U test, while other correlation analyses were performed using Spearman’s rank correlation. Statistical significance was defined as *p* < 0.05, and all analyses were conducted in RStudio (v4.4.1).

## 5. Conclusions

Overall, our findings indicate that upregulated TIGIT expression may be associated with MSI status and *BRAF* mutations in CRC tumors. TIGIT and CD155 protein levels were significantly elevated in CRC tumors and did not show significant associations with the analyzed clinicopathological parameters, including TNM stage. Cytokine profiling of the CRC TME identified IFN-g as a key immune marker associated with TIGIT/CD155 levels.

## Figures and Tables

**Table 1:** Patient characteristics.

	Female	Male	All
Age	66.5 ± 9.64	65.0 ± 8.75	65 ± 9.22
Primary localization of CRC tumor			
Left-side tumor	43	51	94 (71.76%)
Right-side tumor	18	18	36 (27.48%)
T parameter			
T1	2	5	7 (5.34%)
T2	12	9	21 (16.03%)
T3	41	46	87 (66.41%)
T4	8	8	16 (12.21%)
N parameter			
N0	25	30	55 (41.98%)
N1	26	28	54 (41.22%)
N2	11	11	22 (16.79%)
M parameter			
M0	55	56	111 (84.73%)
M1	7	13	20 (15.27%)
TNM Stage			
I	10	9	19 (14.50%)
II	15	18	33 (25.19%)
III	30	30	60 (45.80%)
IV	7	12	19 (14.50%)
Histological Grading			
High	10	12	22 (16.79%)
Low	52	38	109 (83.21%)
MSI Status (*n* = 79)			
MSS tumors	30	35	65 (82.28%)
MSI tumors	9	5	14 (17.72%)

**Table 2:** *KRAS*, *NRAS*, *BRAF*, *PIK3CA*, and *AKT1* gene mutation frequencies in the analyzed subgroup of CRC tumors.

Gene	Mutation Status		Percent (%)	
*n* = 104	Wild-Type	Mutant	Percent Among Mutation of the One Gene	Percent Among All Groups
*KRAS*	66	38		36.53%
*KRAS-117-STATUS*	100	4	10.52%	3.84%
*KRAS-12/13-STATUS*	75	29	76.31%	27.88%
*KRAS-59-STATUS*	101	3	7.89%	2.88%
*KRAS-146-STATUS*	102	2	5.26%	1.92%
*KRAS-61-STATUS*	101	3	7.89%	2.88%
*NRAS*	91	13		12.50%
*NRAS-12-13-STATUS*	96	8	61.54%	7.69%
*NRAS-61-STATUS*	99	5	38.46%	4.80%
*PIK3CA*	97	7		6.73%
*PIK3CA 542/545*	99	6	85.71%	5.77%
*PIK3CA 1047*	103	1	14.28%	0.961%
*BRAF*	97	7		6.73%
*AKT*	103	1		0.961%
Combined gene mutations		9		9.615%
*KRAS + NRAS*		3		2.88%
*KRAS + PIK3CA*		4		3.846%
*KRAS + BRAF*		1		0.961%
*KRAS + AKT1*		1		0.961%
*KRAS + NRAS + BRAF*		1		0.961%

**Table 3:** Eigenvalues and the proportion of variance explained by the three extracted principal components from the PCA for T cell-mediated immunity set of cytokines.

	Eigenvalue	Variance (%)	Cumulative Variance (%)
PCA Factor 1	4.81902287	68.8431839	68.84318
PCA Factor 2	0.86690436	12.3843480	81.22753
PCA Factor 3	0.69758966	9.9655665	91.19310

**Table 4:** Varimax-rotated factor loadings for the three principal components, with corresponding variable coordinates for positive regulation of T cell-mediated immunity set of cytokines.

Variable	Factor 1	Factor 2	Factor 3
T cell-mediated immunity set of cytokines			
IFN-g	0.4719451	−0.86373493	0.09534726
IL-4	0.9027548	0.10977010	0.25980941
GM-CSF	0.8704450	0.20362505	−0.15031801
TNF-a	0.9181357	0.12654604	0.23385815
IL-10	0.8913616	−0.00496560	0.35402042
IL-2	0.9208311	0.15375380	−0.26852000
IL-5	0.7336358	−0.16635779	−0.58845479

**Table 5:** Eigenvalues and the proportion of variance explained by the three extracted principal components from the PCA for Type II interferon signaling set of cytokines.

	Eigenvalue	Variance (%)	Cumulative Variance (%)
PCA Factor 1	2.4060539	48.121078	48.12108
PCA Factor 2	1.1262466	22.524933	70.64601
PCA Factor 3	0.7809113	15.618226	86.26424

**Table 6:** Varimax-rotated factor loadings for the three principal components, with corresponding variable coordinates for Type II interferon signaling set of cytokines.

Variable	Factor 1	Factor 2	Factor 3
Type II interferon signaling set of cytokines			
IFN-g	0.6171093	0.6519835	−0.1687242
IL-1b	0.7696256	0.1599269	−0.4608628
IP-10	0.7193624	−0.4800544	0.3448535
MIG	0.6724328	−0.5876307	−0.2794389
IFN-a2	0.6806309	0.3159515	0.5856952

**Table 7:** Hotspot variants identified by the RT-PCR assay in *KRAS*, *NRAS*, *BRAF*, *PIK3CA*, and *AKT1*, including exon coordinates, amino acid changes, corresponding nucleotide substitutions, and the associated COSMIC IDs.

Gene	Exon	Amino Acid Change	Nucleotide Change	Cosmic ID
*KRAS*	2	G12A	c.35G>C	522
		G12D	c.35G>A	521
		G12R	c.34G>C	518
		G12C	c.34G>T	516
		G12S	c.34G>A	517
		G12V	c.35G>T	520
		G13D	c.38G>A	532
	3	A59T	c.175G>A	546
		A59E	c.176C>A	547
		A59G	c.176C>G	28518
		Q61H	c.183A>C	554
		Q61H	c.183A>T	555
		Q61L	c.182A>T	553
		Q61R	c.182A>G	552
	4	K117N	c.351A>C	19940
		K117N	c.351A>T	28519
		K117R	c.350A>G	4696722
		K117E	c.349A>G	-
		A146T	c.436G>A	19404
		A146P	c.436G>C	19905
		A146V	c.437C>T	19900
*NRAS*	2	G12D	c.35G>A	564
		G12S	c.34G>A	563
		G12C	c.34G>T	562
		G13R	c.37G>C	569
		G13V	c.38G>T	574
	3	A59T	c.175G>A	578
		A59D	c.176C>A	253327
		Q61K	c.181C>A	580
		Q61L	c.182A>T	583
		Q61R	c.182A>G	584
		Q61H	c.183A>C	586
		Q61H	c.183A>T	585
	4	K117R	c.350A>G	-
		A146T	c.436G>A	27174
*BRAF*	15	V600E	c.1799T>A	476
		V600E2	c.1799-1800TG>AA	-
		V600D	c.1799-1800TG>AT	477
		V600K	c.1798-1799GT>AA	473
*PIK3CA*	9	E542K	c.1624G>A	760
		E545K	c.1633G>A	763
		E545Q	c.1633G>C	27133
	20	H1047R	c.3140A>G	775
		H1047L	c.3140A>T	776
*AKT1*	4	E17K	c.49G>A	33765

**Table 8:** Cytokine, chemokine, and growth-factor sets were mapped to the relevant Gene Ontology (GO) terms and annotated using KEGG and WikiPathways (WP) resources.

Process Name	Cytokines Involved	Origin
Positive regulation of immune system process	MIF, SCF, MCP1, SDF-1a, VEGFA, MCP3, MCSF, MIP-1a, IL-1a, IL-18, IL-6, RANTES, IL-5, TNF-b, LIF, IL-2, IL-1b, IL-7, IFN-g, IL-13, TNF-a, IL-10, IL-8, IL-4, IP-10, IL-15, IL-2Ra, IL-16, CTACK, IL-12p40, MIP-1b, IL-17	GO
Chemokine signaling pathway	IL-8, MCP1, SDF-1a, GRO-a, IP-10, RANTES, MIP-1a, CTACK, Eotaxin, MCP3, MIP-1b	KEGG
Positive regulation of lymphocyte migration	IP-10, SDF-1a, MIP-1a, CTACK, MIP-1b, MCP3, RANTES	GO
Macrophage chemotaxis	MCP1, IL-8, Eotaxin, MIG, IP-10, GRO-a, MIP-1b, MCP3, RANTES, IL-1b	GO
PI3K-Akt signaling pathway	IL-2Ra, bNGF, IL-2, IL-3, IL-4, SCF, MCSF, IFN-a2, HGF, G-CSF, IL-7, PDGF-bb, BasicFGF, IL-6, VEGFA	KEGG
Leukocyte activation	IL-4, IL-15, IFN-g, SCF, IL-2Ra, IL-8, MCSF, IL-13, IL-18, MIP-1a, RANTES, IL-10, GM-CSF, IL-9, IL-7, IFN-a2, IL-2, IL-6, TNF-a	GO
Inflammatory response	IL-9, CTACK, Eotaxin, MCP1, IFN-a2, IL-1Ra, IL-2Ra, IFN-g, IL-15, IL-1a, IL-6, IL-17, IL-4, MCP3, MIP-1a, IL-18, CTACK, MIF, TNF-a, RANTES, MCSF, MIG, IL-1b, IL-5, IL-10, IL-8, IL-13, IP-10, MIP-1b	GO
Interleukin-10 signaling	MCP1, MCSF, IL-8, IL-18, IL-6, GM-CSF, LIF, IL-10, IL-1Ra, IL-1a, IP-10, GRO-a, MIP-1a, IL-1b, MIP-1b, G-CSF, RANTES, TNF-a	KEGG
NOD-like receptor signaling pathway	IL-8, TNF-a, MCP1, IFN-a2, GRO-a, IL-6, IL-1b, RANTES, IL-18	KEGG
Positive regulation of T cell-mediated immunity	IL-4, IL-6, IL-1b, IL-18, IL-12p40, IL-12p70	GO
IL-17 signaling	MCP1, IL-8, IL-1b, IL-4, IL-5, IL-6, IL-13, IL-17, GM-CSF, IFN-g, G-CSF, TNF-a, MCP3, Eotaxin, GRO-a, IP-10	KEGG
Positive regulation of cytokine production	IL-9, IL-12p70, GM-CSF, IL-10, HGF, IL-2, IL-15, IL-1b, IL-18, IFN-g, IL-7, IL-4, TNF-a, IL-17, MIF, TNF-b, IL-16, IL-13, MIP-1a, IL-1a, IL-6	GO
Type II interferon signaling	IFN-g, IL-1b, IP-10, MIG, IFN-a2	WP
IL-18 signaling pathway	MCP1, IFN-g, IL-12p40, IL-1b, IL-9, IL-18, IL-13, IL-8, IL-2Ra, IL-6, TNF-a, IL-10, RANTES, MIP-1a, MIP-1b, SCF	WP
Signal transduction through IL1R	IL-1Ra, IL-1a, IL-1b, IL-6, TNF-a	WP

## Data Availability

GSEA data: Dampier CH (2020). “FieldEffectCrc: Tumor, tumor-adjacent normal, and healthy colorectal transcriptomes as SummarizedExperiment objects”. https://bioconductor.org/packages/release/data/experiment/html/FieldEffectCrc.html (accessed on 10 December 2025). The original contributions presented in this study are included in the article/supplementary material. Further inquiries can be directed to the corresponding author(s).

## Supplementary Materials

The following supporting information can be downloaded at https://www.mdpi.com/article/10.3390/ijms27020937/s1.

## Abbreviations

The following abbreviations are used in this manuscript:

AKT1	AKT serine/threonine kinase 1
BRAF	B-Raf proto-oncogene, serine/threonine kinase
CIMP	CpG island methylator phenotype
CMS	Consensus Molecular Subtypes
COAD	Colon adenocarcinoma (TCGA)
CRC	Colorectal cancer
CYT	Cytolytic activity
DC(s)	Dendritic cell(s)
DESeq2	Differential expression analysis package (DESeq2)
DFS	Disease-free survival
dMMR	Deficient mismatch repair
EGFR	Epidermal growth factor receptor
ELISA	Enzyme-linked immunosorbent assay
EMT	Epithelial–mesenchymal transition
ERK	Extracellular signal-regulated kinase
FDR	False discovery rate
FFPE	Formalin-fixed, paraffin-embedded
fgsea	Fast gene set enrichment analysis (R package)
GSEA	Gene set enrichment analysis
HRP	Horseradish peroxidase
ICI(s)	Immune checkpoint inhibitor(s)
IHC	Immunohistochemistry
ILC(s)	Innate lymphoid cell(s)
iNKT	Invariant natural killer T cell
IFN	Interferon
IFN-a2	Interferon alpha 2
IFN-g	Interferon gamma
KRAS	Kirsten rat sarcoma viral oncogene homolog
LIF	Leukemia inhibitory factor
LMM	Linear mixed(-effects) model
MAIT	Mucosa-associated invariant T cell
MAPK	Mitogen-activated protein kinase
MCP-1	Monocyte chemoattractant protein 1 (CCL2)
MCP-3	Monocyte chemoattractant protein 3 (CCL7)
M-CSF	Macrophage colony-stimulating factor
MEK	MAPK/ERK kinase
MIF	Macrophage migration inhibitory factor
MIG	Monokine induced by gamma interferon (CXCL9)
MMR	Mismatch repair
mRNA	Messenger RNA
mTORC1	Mechanistic target of rapamycin complex 1
MSS	Microsatellite stable
MSI	Microsatellite instability
MSigDB	Molecular Signatures Database
NF-κB	Nuclear factor kappa B
NK cell(s)	Natural killer cell(s)
NES	Normalized enrichment score
NRAS	Neuroblastoma RAS viral oncogene homolog
OS	Overall survival
PDGF-BB	Platelet-derived growth factor BB
PIK3CA	Phosphatidylinositol-4,5-bisphosphate 3-kinase catalytic subunit alpha
pMMR	Proficient mismatch repair
PVR/CD155	Poliovirus receptor (CD155)
qPCR	Quantitative polymerase chain reaction
RANTES	Regulated upon activation, normal T cell expressed and secreted (CCL5)
READ	Rectal adenocarcinoma (TCGA)
SCF	Stem cell factor (KIT ligand)
SCGF-B	Stem cell growth factor beta (CLEC11A)
SDF-1a	Stromal cell-derived factor 1 alpha (CXCL12)
TCGA	The Cancer Genome Atlas
Tfh	T follicular helper cell
TIGIT	T cell immunoreceptor with Ig and ITIM domains
TIL(s)	Tumor-infiltrating lymphocyte(s)
TME	Tumor microenvironment
TNF-a	Tumor necrosis factor alpha
TNF-b	Tumor necrosis factor beta (lymphotoxin-a)
TNM	Tumor–node–metastasis classification
TRAIL	TNF-related apoptosis-inducing ligand
Treg(s)	Regulatory T cell(s)
Tr1	Type 1 regulatory T cell
VEGF-A	Vascular endothelial growth factor A
GBM	Glioblastoma multiforme (glioblastoma)
