# Supplementary material for: Association of TIGIT and CD155 with KRAS, NRAS, BRAF, PIK3CA, and AKT Gene Mutations, MSI Status, and Cytokine Profiles in Colorectal Cancer
Source: Int J Mol Sci. 2026 Jan 17;27(2):937. doi: 10.3390/ijms27020937 (PMC12841434; doi:10.3390/ijms27020937)
Supplement: Supplementary file 1 [file ijms-27-00937-s001.zip › ijms-4074816-supplementary.pdf]

International Journal of *Molecular Sciences*

Article

Association of TIGIT and CD155 with KRAS, NRAS, BRAF, PIK3CA, and AKT Gene Mutations, MSI Status, and Cytokine Profiles in Colorectal Cancer

Supplementary Materials

Błażej Ochman [1], Piotr Limanówka [1], Sylwia Mielcarska [1], Agnieszka Kula [2], Miriam Dawidowicz [2], Dorota Hudy [1], Monika Szrot [3], Jerzy Piecuch [3], Zenon Czuba [4], Dariusz Waniczek [2], Elżbieta Świętochowska [1]

[1] Department of Medical and Molecular Biology, Faculty of Medical Sciences in Zabrze, Medical University of Silesia, 19 Jordana, 41-808 Zabrze, Poland; d201228@365.sum.edu.pl (B.O.); s s82955@365.sum.edu.pl (P.L.); d201109@365.sum.edu.pl (S.M.); dorota.hudy@sum.edu.pl (D.H.)

[2] Department of Oncological Surgery, Faculty of Medical Sciences in Zabrze, Medical University of Silesia, 41-808 Katowice, Poland; d201070@365.sum.edu.pl (A.K.); d201069@365.sum.edu.pl (M.D.); dwaniczek@sum.edu.pl (D.W.)

[3] Department of General and Bariatric Surgery and Emergency Medicine in Zabrze, Faculty of Medical Sciences in Zabrze, Medical University of Silesia, 10 Marii Curie-Skłodowskiej, 41-800 Zabrze, Poland; mszrot@sum.edu.pl (M.S.); jpiecuch@sum.edu.pl (J.P.)

[4] Department of Microbiology and Immunology, Faculty of Medical Sciences in Zabrze, Medical University of Silesia, 19 Jordana, 41-808 Zabrze, Poland; zczuba@sum.edu.pl

* Correspondence: d201228@365.sum.edu.pl

Academic Editor: Firstname Lastname

Received: date
Revised: date
Accepted: date
Published: date

Citation: To be added by editorial staff during production.

Copyright: © 2025 by the authors. Submitted for possible open access publication under the terms and conditions of the Creative Commons Attribution (CC BY) license (https://creativecommons.org/licenses/by/4.0/).

Protein	Fixed Effect	Estimate (β)	Standard Error	Degrees of Freedom	p-Value
TIGIT	Intercept (Margin)	-0.5823	0.0099	204.08	< 0.0001 (***)
TIGIT	Tumor vs. Margin	0.0803	0.0097	130.00	< 0.0001 (***)
CD155	Intercept (Margin)	-1.1739	0.0080	248.17	< 0.0001 (***)
CD155	Tumor vs. Margin	0.1052	0.0101	128.94	< 0.0001 (***)

Table S1A. Linear mixed-effects model results comparing TIGIT and CD155 expression in tumor tissue and matched surgical margins - Fixed Effects.

Protein	Random Effect	Variance	Standard Deviation
TIGIT	Patient ID	0.0067	0.0820
TIGIT	Residual	0.0061	0.0782
CD155	Patient ID	0.0018	0.0419
CD155	Residual	0.0067	0.0816

Table S1B. Linear mixed-effects model results comparing TIGIT and CD155 expression in tumor tissue and matched surgical margins - Random Effects.

	Tumor grading	Primary tumor localization	MSI status
	p-value	p-value	p-value
TIGIT	0.5511	0.3077	0.0305 *
CD155	0.09478	0.872	0.7628

Table S2. TIGIT and CD155 proteins concentration and tumor grading, primary tumor localization (Left-Side Tumors vs. Right-Side Tumors), and MSI status. p-value was derived from the Mann–Whitney U test for TIGIT and CD155 proteins concentration. * p-value < 0.05.

	T parameter		N parameter		M parameter	Tumor Stage		Tumor Infiltrated Lymphocytes (TILs)	
	p-value	tau	p-value	tau	p-value	p-value	tau	p-value	tau
TIGIT	0.2173	0.0851	0.6194	-0.03408	0.315	0.5221	0.04279	0.8076	0.02112
CD155	0.09546	0.1155	0.05454	-0.1325	0.5842	0.4708	-0.04841	0.5337	0.05425

Table S3. TIGIT and CD155 protein concentration and TNM scale parameters, tumor stage, and TILs. Kendall's Tau rank correlation coefficient p-value and Tau for T and N parameters, tumor stage, and TILs for TIGIT and CD155 expression. p-value for the M parameter was derived from the Mann–Whitney U test.

Gene	TIGIT	CD155
n = 106	p-value	p-value
KRAS	0.8832	0.6692
KRAS-117-STATUS	0.1916	0.9252
KRAS-12/13-STATUS	0.3116	0.6732
KRAS-59-STATUS	0.5497	0.9687
KRAS-146-STATUS	0.2768	0.04857 *
KRAS-61-STATUS	0.3365	0.6732
NRAS	0.781	0.6406
NRAS-12-13-STATUS	0.8582	0.6887
NRAS-61-STATUS	0.8419	0.8298
PIK3CA	0.9373	0.387
BRAF	0.006416 *	0.1856
AKT	0.355	0.4292
Multimutated tumor group vs non-mutant tumor group	0.7434	0.2974

Table S4. KRAS, NRAS, BRAF, PIK3CA, AKT gene mutations and TIGIT, CD155 protein expression. Multimutated tumor group contain tumors with more than one gene mutation. U-Mann-Whitney test p-value results. * $p < 0.05$.

Cytokine	Spearman R TIGIT	p-value TIGIT	Spearman R CD155	p-value CD155
IFN-g	0.473	0.0351 *	0.532	0.0157 *
IL-1Ra	0.420	0.0649	0.505	0.0231*
HGF	-0.415	0.0687	-0.267	0.2550
IL-12p40	-0.381	0.0972	-0.202	0.3920
SCGF-b	-0.328	0.1580	-0.120	0.6150
IL-13	-0.324	0.1630	-0.130	0.5850
MCSF	0.264	0.2610	0.483	0.0309 *
TRAIL	0.257	0.2740	0.486	0.0300 *
SCF	-0.220	0.3520	-0.032	0.8920
PDGF-bb	0.199	0.4010	0.539	0.0142 *
IL-6	-0.185	0.4350	-0.047	0.8430
GM-CSF	-0.170	0.4740	0.098	0.6810
IL-16	-0.157	0.5080	-0.079	0.7400
TNF-a	-0.156	0.5100	0.184	0.4380
IL-12p70	-0.154	0.5170	0.173	0.4660
GRO-a	-0.150	0.5270	0.050	0.8350
bNGF	-0.135	0.5690	0.120	0.6150
IL-1a	-0.127	0.5930	0.211	0.3720
IL-1b	0.121	0.6110	0.361	0.1180
CTACK	-0.119	0.6190	0.189	0.4260
MIF	0.117	0.6240	0.249	0.2900
TNF-b	0.117	0.6240	0.365	0.1130
IL-4	-0.116	0.6270	0.131	0.5820
IL-18	-0.115	0.6290	0.192	0.4180
BasicFGF	0.102	0.6680	0.139	0.5580
IL-2Ra	0.100	0.6750	0.361	0.1180
MIP-1a	-0.097	0.6840	0.083	0.7290
SDF-1a	-0.087	0.7140	0.254	0.2800
IL-3	-0.080	0.7380	0.141	0.5540
MIG	0.077	0.7450	0.245	0.2970
IL-10	-0.066	0.7820	0.222	0.3480
IL-9	0.064	0.7890	0.339	0.1440
RANTES	0.059	0.8060	0.275	0.2400
IFN-a2	0.041	0.8620	0.322	0.1660
MCP1	-0.035	0.8850	0.136	0.5670
IL-15	-0.034	0.8870	0.145	0.5430
VEGFA	-0.029	0.9020	0.146	0.5390
IL-8	-0.023	0.9220	0.302	0.1960
MCP3	0.021	0.9300	0.279	0.2330
LIF	0.016	0.9470	0.261	0.2660
G-CSF	-0.015	0.9500	0.154	0.5180
IP-10	-0.015	0.9500	0.199	0.4010
IL-5	0.011	0.9620	0.129	0.5860
Eotaxin	-0.008	0.9750	0.186	0.4320
IL-2	-0.005	0.9820	0.226	0.3380
MIP-1b	0.003	0.9900	0.291	0.2130
IL-17	-0.002	0.9920	0.196	0.4080
IL-7	0.000	0.9990	0.072	0.7630

Table S5. Spearman correlations between TIGIT or CD155 (PVR) expression and cytokines, chemokines and growth factors levels in CRC tissue homogenates. * p-value < 0.05.

	Pathway	NES	pval	padj	Size	Enrichment
1	HALLMARK_INTERFERON_GAMMA_RESPONSE	3,12	0,000194	0,000466	199	Up-regulated
2	HALLMARK_INFLAMMATORY_RESPONSE	3,11	0,000195	0,000466	200	Up-regulated
3	HALLMARK_ALLOGRAFT_REJECTION	3,02	0,000195	0,000466	200	Up-regulated
4	HALLMARK_IL6_JAK_STAT3_SIGNALING	2,8	0,0002	0,000466	87	Up-regulated
5	HALLMARK_INTERFERON_ALPHA_RESPONSE	2,76	0,0002	0,000466	97	Up-regulated
6	HALLMARK_TNFA_SIGNALING_VIA_NFKB	2,69	0,000194	0,000466	199	Up-regulated
7	HALLMARK_EPITHELIAL_MESENCHYMAL_TRANSITION	2,52	0,000195	0,000466	200	Up-regulated
8	HALLMARK_COMPLEMENT	2,49	0,000195	0,000466	200	Up-regulated
9	HALLMARK_IL2_STAT5_SIGNALING	2,41	0,000194	0,000466	199	Up-regulated
10	HALLMARK_KRAS_SIGNALING_UP	2,3	0,000195	0,000466	200	Up-regulated
11	HALLMARK_APOPTOSIS	1,95	0,000198	0,000466	160	Up-regulated
12	HALLMARK_ANGIOGENESIS	1,94	0,000204	0,000466	36	Up-regulated
13	HALLMARK_UV_RESPONSE_DN	1,89	0,000197	0,000466	143	Up-regulated
14	HALLMARK_APICAL_JUNCTION	1,72	0,000195	0,000466	200	Up-regulated
15	HALLMARK_COAGULATION	1,71	0,000196	0,000466	138	Up-regulated
16	HALLMARK_HYPOXIA	1,7	0,000195	0,000466	200	Up-regulated
17	HALLMARK_UNFOLDED_PROTEIN_RESPONSE	-1,85	0,000201	0,000466	113	Down-regulated
18	HALLMARK_DNA_REPAIR	-2,08	0,000204	0,000466	150	Down-regulated
19	HALLMARK_E2F_TARGETS	-2,12	0,000205	0,000466	200	Down-regulated
20	HALLMARK_MYC_TARGETS_V2	-2,15	0,000199	0,000466	58	Down-regulated
21	HALLMARK_OXIDATIVE_PHOSPHORYLATION	-2,63	0,000205	0,000466	200	Down-regulated
22	HALLMARK_MYC_TARGETS_V1	-2,67	0,000205	0,000466	200	Down-regulated
23	HALLMARK_TGF_BETA_SIGNALING	1,82	0,000403	0,000876	54	Up-regulated
24	HALLMARK_ANDROGEN_RESPONSE	1,54	0,0042	0,00839	101	Up-regulated
25	HALLMARK_KRAS_SIGNALING_DN	1,4	0,00546	0,0105	200	Up-regulated
26	HALLMARK_PEROXISOME	-1,4	0,0214	0,0382	103	Down-regulated

Table S6. Gene Set Enrichment Analysis (GSEA) for TIGIT high vs low expression. The Hallmark gene sets significantly enriched in the TIGIT high vs low expression. *GSEA (Gene Set Enrichment Analysis); NES (Normalized Enrichment Score); pval (nominal p-value); padj (FDR-adjusted p-value).*

	Pathway	NES	pval	padj	Size	Enrichment
1	HALLMARK_KRAS_SIGNALING_UP	-1,52	0,0001	0,000507	200	Down-regulated
2	HALLMARK_ADIPOGENESIS	-1,54	0,0001	0,000507	200	Down-regulated
3	HALLMARK_KRAS_SIGNALING_DN	-1,54	0,0001	0,000507	200	Down-regulated
4	HALLMARK_HEME_METABOLISM	-1,55	0,000101	0,000507	197	Down-regulated
5	HALLMARK_COMPLEMENT	-1,62	0,0001	0,000507	200	Down-regulated
6	HALLMARK_INTERFERON_GAMMA_RESPONSE	-1,64	0,0001	0,000507	199	Down-regulated
7	HALLMARK_UV_RESPONSE_DN	-1,64	0,000101	0,000507	143	Down-regulated
8	HALLMARK_ALLOGRAFT_REJECTION	-1,64	0,0001	0,000507	200	Down-regulated
9	HALLMARK_INFLAMMATORY_RESPONSE	-1,65	0,0001	0,000507	200	Down-regulated
10	HALLMARK_MYOGENESIS	-1,83	0,0001	0,000507	199	Down-regulated
11	HALLMARK_FATTY_ACID_METABOLISM	-1,55	0,000202	0,000918	157	Down-regulated
12	HALLMARK_BILE_ACID_METABOLISM	-1,59	0,000309	0,00129	111	Down-regulated
13	HALLMARK_MYC_TARGETS_V2	3,48	0,00129	0,00497	58	Up-regulated
14	HALLMARK_UNFOLDED_PROTEIN_RESPONSE	2,58	0,00366	0,0109	113	Up-regulated
15	HALLMARK_APICAL_JUNCTION	-1,37	0,00372	0,0109	200	Down-regulated
16	HALLMARK_EPITHELIAL_MESENCHYMAL_TRANSITION	-1,37	0,00352	0,0109	200	Down-regulated
17	HALLMARK_IL2_STAT5_SIGNALING	-1,37	0,00372	0,0109	199	Down-regulated
18	HALLMARK_DNA_REPAIR	2,21	0,0082	0,0216	150	Up-regulated
19	HALLMARK_PANCREAS_BETA_CELLS	-1,52	0,0102	0,0256	40	Down-regulated
20	HALLMARK_MYC_TARGETS_V1	3,67	0,0222	0,0383	200	Up-regulated
21	HALLMARK_E2F_TARGETS	3,63	0,0222	0,0383	200	Up-regulated
22	HALLMARK_G2M_CHECKPOINT	3,17	0,0222	0,0383	200	Up-regulated
23	HALLMARK_MTORC1_SIGNALING	2,62	0,0222	0,0383	200	Up-regulated
24	HALLMARK_GLYCOLYSIS	1,44	0,0222	0,0383	200	Up-regulated
25	HALLMARK_UV_RESPONSE_UP	1,21	0,02	0,0383	157	Up-regulated
26	HALLMARK_WNT_BETA_CATENIN_SIGNALING	1,44	0,0262	0,0437	42	Up-regulated

Table S7. Gene Set Enrichment Analysis (GSEA) for CD155 high vs low expression. The Hallmark gene sets significantly enriched in the CD155 high vs low expression. *GSEA (Gene Set Enrichment Analysis); NES (Normalized Enrichment Score); pval (nominal p-value); padj (FDR-adjusted p-value).*

Disclaimer/Publisher's Note: The statements, opinions and data contained in all publications are solely those of the individual author(s) and contributor(s) and not of MDPI and/or the editor(s). MDPI and/or the editor(s) disclaim responsibility for any injury to people or property resulting from any ideas, methods, instructions or products referred to in the content.
